# Coaching-Driven Digital Literacy for Older Adults: Results from AGAPE Ecosystem Pilot Study

**DOI:** 10.3390/geriatrics11050124

**Published:** 2026-09-06

**Authors:** Matei Teodorescu, Chiara Pedrini, Ileana Ciobanu, Andreea Georgiana Marin, Emanuela Elena Mihai, Sabrina Tazzari, Maria Luigia del Vicario, Jensen Selwyn Joymangul, Ana Goreti Oliveira, Elena Tamburini, Francesco Agnoloni, Francesca Cecchi, Mihai Berteanu

**Affiliations:** 1 Discipline of Physical and Rehabilitation Medicine—Elias University Emergency Hospital, Carol Davila University of Medicine, 011461 Bucharest, Romania; matei.teodorescu@umfcd.ro (M.T.); andreea.budrica@gmail.com (A.G.M.); emanuela-elena.mihai@umfcd.ro (E.E.M.); mihai.berteanu@umfcd.ro (M.B.); 2 IRCCS Fondazione Don Carlo Gnocchi, 50143 Florence, Italy; stazzari@dongnocchi.it (S.T.); mdelvicario@dongnocchi.it (M.L.d.V.); fcecchi@dongnocchi.it (F.C.); 3 Luxembourg Institute of Science and Technology, L-4362 Esch-sur-Alzette, Luxembourg; jensen.joymangul@list.lu; 4 Cáritas Diocesana de Coimbra, 3030-382 Coimbra, Portugal; anagoliveira@caritascoimbra.pt; 5 Medea S.r.l., 50144 Florence, Italy; e.tamburini@medeaproject.eu (E.T.); f.agnoloni@medeaproject.eu (F.A.); 6 Department of Experimental and Clinical Medicine, University of Florence, 50143 Florence, Italy

**Keywords:** digital health equity, active and assisted living, digital literacy, telehealth, ageing, coaching, co-creation, inclusion

## Abstract

**Introduction:** Rapid technological development requires continuous learning and adaptation, yet many older adults (OAs) face difficulties using digital technologies. The AGAPE project (Active aGeing And Personalised service Ecosystem) aimed to promote active and healthy ageing (AHA) among adults aged 65+ by supporting independent functioning reducing caregiver burden, and improving eHealth literacy through personalized, user-friendly digital services co-created and tested in Italy, Portugal, and Romania. **Objective:** AGAPE pilot phase evaluated the effectiveness and usability of innovation adoption strategies designed to improve technology acceptance, digital literacy, and behavioural change among older adults and caregivers. This study examined participants’ perceptions of digital technologies and self-reported digital efficacy during a multicomponent intervention combining repeated technology use, personalized coaching, digital literacy and health education, technical assistance, and support from informal and formal caregivers. **Material and Methods:** The multiple-sites uncontrolled pilot study combined home-based use of the AGAPE digital technologies with long-term combined personalized support. Data were collected at baseline (T0), intermediate (T1), and final (T2) assessments using the System Usability Scale (SUS), technostress questionnaire, digital adoption questionnaire (TAM-derived), EQ-5D-5L, CarerQoL-7D, and the UCLA Loneliness Scale, complemented by participant feedback, technology usage, and activity data. **Results:** The study included 217 participants, of whom 146 were OAs. Over the course of the intervention, participants showed reductions in technostress and improvements in perceived digital efficacy, although the magnitude and direction of change varied across pilot sites. The findings also indicated that contextual and social factors contributed to technology adoption and engagement. **Conclusions:** Participation in the multicomponent AGAPE intervention was associated with positive longitudinal changes in technology-related perceptions, technostress, and digital self-efficacy. The findings support the feasibility and potential of combining digital technologies with personalized coaching and broader interpersonal support to facilitate technology adoption among older adults. However, given the uncontrolled study design and the integrated nature of the intervention, the observed changes cannot be attributed independently to coaching or to any single intervention component.

## 1. Introduction

As populations worldwide continue to age, supporting older adults (OAs) in maintaining active, healthy, and fulfilling lives has become an important societal priority. This demographic transition places increasing pressure on health and social care systems, highlighting the need for scalable, person-centred solutions that improve quality of life while supporting the long-term sustainability of care services. The European Commission has promoted this objective through research and funding programmes such as Horizon 2020 [1] and initiatives including the European Innovation Partnership on active and healthy ageing (EIP on AHA) [2]. These initiatives have encouraged the development, transferability, and wider implementation of digital health solutions designed to support healthy ageing.

Consistent with this European agenda, the WHO-supported Decade of Healthy Ageing promotes a life-course and multisectoral approach to ageing that contributes to the 2030 Agenda and the Sustainable Development Goals [3,4,5]. Its priorities include changing societal attitudes towards ageing, developing age-friendly communities, providing person-centred and integrated health and social care, and ensuring access to long-term care when required. Because older adults may encounter systemic barriers such as ageism, ableism, and mentalism, care systems must be organized around dignity, autonomy, inclusion, and coordinated support [3].

In an increasingly digital society, acquiring digital skills and adapting everyday behaviours to incorporate technology are becoming essential for maintaining well-being, accessing health and social care services and health information, supporting communication, and enabling independent living [6,7]. Nevertheless, many older adults experience difficulties in adopting digital technologies. Identified barriers include frailty, age-related sensory and motor impairments, limited social support, negative perceptions of technology, and insufficient access to appropriate resources [8]. Evidence suggests that user-friendly and personalized technologies may support well-being, health self-management, healthier lifestyles, and social communication, particularly when their design and implementation reflect users’ abilities and needs [9,10].

Within this context, the AGAPE project (Active aGeing And Personalised service Ecosystem) was developed to implement and evaluate innovative, personalized services for active and healthy ageing through digital technologies and wearable sensors. The project sought to reduce the eHealth literacy gap, support behavioural adaptation, and alleviate caregiver burden by promoting the meaningful adoption of technology among older adults and their support networks. Technology use was facilitated through individualized coaching, technical guidance, and the involvement of family members and health and social care professionals.

AGAPE provided a set of co-created services tailored to individual needs, including digital literacy training, lifestyle and health monitoring, and support for social participation. These services were delivered through dedicated digital tools with the assistance of trained AGAPE coaches. This approach is consistent with evidence indicating that supported digital learning and reverse mentoring may strengthen self-efficacy, improve digital literacy, and promote more positive perceptions of ageing [11].

The AGAPE model was grounded in person-centred care, co-creation, and the active involvement of multiple stakeholder groups. Primary users were older adults; secondary users included informal family caregivers and formal caregivers from health and social care services; and tertiary users comprised representatives of health and social care organizations.

The objective of this study was to evaluate longitudinal changes in participants’ perceptions of digital technologies, digital adoption, technostress, and usability during a multicomponent intervention combining repeated technology use, personalized coaching, and support from informal and formal caregivers across three European pilot sites.

## 2. Materials and Methods

The pilot is a non-control intra-individual longitudinal impact study representing the 4th phase in the co-creation process in AGAPE AAL Project (Active Assisted Living), as detailed in the previously published paper describing the results of co-creation and pre-validation activities in AGAPE [11].

**The AGAPE service scenarios.** AGAPE delivered personalized digital services using wearables (Sensoria Health Inc., Redmond, WA, USA, v. 2) and AGAPE Assistant app (delivered by the consortium, v. 1), tailored to older adults’ needs, digital skills, and innovation adoption levels, aiming to promote active ageing, lifestyle monitoring, and eHealth literacy. Services were adapted to individual user profiles to ensure accessibility and relevance. Formal caregivers accessed dedicated monitoring and care coordination tools (AGAPE Monitor and AGAPE Eye), while informal caregivers used communication applications—the IoChat app from Senlab—and were often involved in testing technologies to better support older adults.

**The AGAPE coach.** A key component of the methodology was the intervention of the AGAPE coach, a trained healthcare professional who provided personalized support, motivation, and training through in-person or remote interactions. Coaches helped participants understand and use digital technologies, monitored progress, adapted individual goals, and encouraged long-term engagement through tailored feedback and rewarding mechanisms.

User progress and adherence were monitored through the AGAPE Monitor web app developed in AGAPE project, which integrated data from the AGAPE Assistant smartphone app and wearable devices. The platform enabled remote monitoring of physical activity, digital literacy objectives, and personalized user journeys, allowing coaches to tailor support according to participants’ needs and progress.

**The study design** of the pilot phase included enrolling older adults along with their informal and formal caregivers, as well as care organization managers. The AGAPE pilot was designed as a multicomponent intervention rather than as an evaluation of the coaching method per se. Participants received repeated exposure to digital technologies alongside personalized coaching, technical assistance, health and digital literacy education, along with support from informal and formal caregivers. Accordingly, the study design did not allow separation of independent impact of the intervention components.

Inclusion criteria were defined for each stakeholder category and included: (a) for **primary end users—older adults (OAs):** age 65 years or older; ability to provide informed consent; clinically stable; absence of moderate to severe cognitive impairment; (b) for **secondary end users—formal caregivers (FCs):** healthcare and social care professionals effective in managing the care of older adults; (c) for **secondary end users—informal caregivers (ICs):** family members effective in managing the care of older adults; (d) for **tertiary end users:** care organization representatives, local authorities, and municipalities.

The full pilot phase evaluated the impact of the AGAPE platform on the daily lives of older adults, caregivers, and care organizations. The intervention lasted between 4 and 12 months across the participating countries, with adaptations to local implementation schedules and organizational requirements. All end users received training on AGAPE technologies according to their innovation adoption (IA) level.

AGAPE coaches were specifically trained to deliver personalized digital literacy programmes and behavioural change interventions tailored to participants’ background and confidence with technology. Coaching strategies included face-to-face as well as remote support, consisting in positive reinforcement, feedback on physical activity level, and information and education on health, active and healthy ageing and eHealth. The roles and responsibilities of the AGAPE coaches are described in Table A1 in the Appendix A.

### 2.1. Technology

The AGAPE platform evaluated in the pilot study integrated digital solutions developed through a co-creation process and previously validated in living-lab and pre-pilot activities [11]. The platform included a market application for confidential, high privacy communication developed for older adults (IoChat), wearable devices for monitoring physical activity and vital parameters, the AGAPE Assistant smartphone app for data collection and user feedback, and the AGAPE Monitor web application, which enabled remote monitoring by coaches and other formal caregivers. A detailed description of the technological platform has been published previously [5]. The study was based on the hypothesis that AGAPE’s innovation adoption strategies and digital tools can improve quality of life, well-being and technology acceptance among older adults and caregivers, while enhancing the sustainability of digitally enabled care services.

### 2.2. Pilot Phase Data Collection Procedure

The pilot phase followed the AGAPE living-lab and pre-pilot phases and assessed the real-life impact of the platform on behavioural change, innovation adoption, and service usability [8].

Both subjective outcomes (questionnaires and user feedback) and objective measures (technology usage and activity data) were collected to support a comprehensive evaluation of the intervention.

Data were collected at baseline (T0), intermediate assessment (T1), and final evaluation (T2). 

Validated instruments, together with the project-specific DA questionnaire, were selected to provide a comprehensive assessment of the impact of the AGAPE intervention upon the usability and acceptance of the technology, health-related quality of life, caregiver outcomes, and social well-being.

**The assessment toolkit** included the following self-reported questionnaires:

(a) Tools for assessing the participants’ attitude towards AGAPE technology and intervention:
  - The System Usability Scale (SUS) [12], a validated 10-item questionnaire used to assess the perceived usability of digital technologies;
  - The technostress questionnaire [13], a three-dimensional questionnaire used to evaluate stress associated with technology use [14];
  - The digital adoption (DA) questionnaire (see description in the paragraphs below).
(b) Tools for the assessment of the quality of life of older adults and informal caregivers, respectively:
  - The EuroQol five-dimensional five-level questionnaire (EQ-5D-5L) [15], used to measure health-related quality of life of primary users;
  - The Care-Related Quality of Life instrument (CarerQoL-7D) [16], used to assess the quality of life and burden of informal caregivers.
(c) Tool to assess the social dimension of the impact of the intervention:
  - The University of California Los Angeles (UCLA) Loneliness Scale [17], used to evaluate perceived loneliness.
(d) Romanian site research team assessed also the ability and complexity of independent activities of daily living in the older adults participants, using the Functional Activities Scale assessment tool, reported by the clinicians.

The digital adoption (DA) questionnaire, an ad hoc instrument developed for the AGAPE project through a structured adaptation of the Unified Theory of Acceptance and Use of Technology (UTAUT) [18], informed by the technology acceptance model (TAM) [13] and on recent evidence on digital health adoption [19]. The DA questionnaire was developed through a literature review, expert co-design involving clinicians, engineers, and behavioural scientists, and after pilot testing with end users. It assesses core determinants of technology acceptance, including core UTAUT constructs (e.g., effort expectancy, performance expectancy, and social influence), and additional determinants identified as significant in the recent literature on digital health adoption, such as perceived ubiquity, self-efficacy, and privacy concerns, as highlighted by Liu et al. (2022) [20] and Rouidi et al. (2022) [21]. All questions (57) are scored in a 7-level Likert scale and for the analysis the sum of responses for each of the considered domain has been computed to evaluate potential statistical significant shifts from baseline to final assessments.

The subjective assessment was completed with non-structured communication of the research team with study participants.

A non-structured **heuristic evaluation** of the technologies was performed at baseline. It involved older adults, informal caregivers, formal caregivers, and coaches participating in the AGAPE pilot who completed the relevant evaluation procedures. These participants were drawn from the overall pilot sample rather than recruited separately for a qualitative substudy. Free feedback from participants regarding the technology-related unmet needs was collected at the intermediate and final assessments through paper-based structured evaluation forms and participant comments regarding usability, technology-related difficulties, coaching support, and overall experience. The collected information was entered into the SMILE platform. Each participant was identified using a pseudonymized code comprising a site-specific letter and a three-digit number based on recruitment order. Responses were summarized descriptively according to the predefined domains of the heuristic evaluation instrument and, where relevant, by user category. Participant comments were reviewed to provide contextual explanations for the observed ratings and to identify recurrent practical observations. No formal thematic analysis, transcript coding, data-saturation assessment, or inter-coder reliability procedure was undertaken.

### 2.3. Data Processing and Statistical Methods Applied

To collect all data from participants, the SMILE platform (from Medea Srl.) was used. The online platform allowed the creation of individual pseudonymized participant profiles and was set to record item by item answers and to calculate validated scores for the different questionnaires applied. SMILE supports multi-level user profiles, with specific permissions based on roles such as administrators or read-only users. The platform features a modular structure that includes data entry, validation, pre-processing, and export functionalities, allowing structured and traceable data workflows. Hosted on a secure server in Italy, it also includes incremental backup and a disaster recovery policy to ensure data integrity and availability. The platform’s index is available at https://www.smiledatahub.com/index.php (accessed on 1 February 2026).

The pre-processing of the AGAPE pilot-aggregated data involved a comprehensive series of steps to ensure the data was in a suitable format for analysis. These steps were meticulously carried out using R software (version 4.5.1-2025-06-13 ucrt), following the export of data tables from the SMILE dashboard in .xlsx format. Hereafter a list of the completed actions:

Firstly, mandatory variables were specified to maintain consistency and ensure accurate data submissions. These variables included recruitment ID, recruitment date, year of birth, gender, target group, and pilot site. Any missing items for other variables were considered as not available during the analysis phase.

For longitudinal analyses, denominators differed across outcomes because each analysis was based on participants with available data for the corresponding questionnaire and, where applicable, paired assessments with the required minimum interval between baseline and follow-up.

Only participants with paired assessments for the corresponding outcome and a minimum interval of 135 days between baseline and follow-up evaluations were included in baseline–final analyses. Missing data were not imputed, and statistical analyses were performed using the available complete observations for each outcome.

The dataset was subsequently reformatted to ensure compatibility with the R statistical software. Variable types were assigned according to their characteristics, with numeric formats used for continuous variables (e.g., year of birth and questionnaire scores), character formats for participant identifiers and technology-related information, and factor formats for categorical variables. This pre-processing step ensured data consistency and facilitated subsequent statistical analyses. Additional variables were then generated to support the analyses. Participants’ age was calculated by subtracting the year of birth from the year of analysis (2025). Furthermore, categorical variables were standardized by harmonizing category labels and recoding binary variables into descriptive categories, thereby improving data consistency and interpretability.

Some variables required re-grouping into fewer, more populated categories. For instance, the educational level variable, originally organized into nine ISCED levels, was reclassified into three broader categories: low (ISCED levels 0 to 2—up to lower secondary education), medium (ISCED levels 3 and 4—upper secondary education or post-secondary non-tertiary education), and high (ISCED levels 5 to 8—short-cycle tertiary education, Bachelor’s or equivalent level, Master’s or equivalent level, Doctoral or equivalent level).

All information retrieved from the SMILE dashboard, including demographic baseline measurements and survey answers collected longitudinally regarding users’ (both OAs and Carers) QoL, loneliness, technology usability and perceived stress, and digital adoption, were merged into a unified data matrix.

This comprehensive dataset was then ready for analysis using R software (version 4.5.1—13 June 2025 ucrt). The following packages were employed: readxl, tidyr, dplyr, ggplot2, lme4, lmerTest, Epi, emmeans, clustMixType, cluster, and ggcorrplot.

Longitudinal changes across assessment phases (baseline, intermediate, and final) were examined using linear mixed-effects models fitted with the *lmer()* function, with assessment phase as a fixed effect and subject as a random effect to account for repeated measures within individuals [22] (following the framework of Laird & Ware [23]). Where distributional assumptions were not met, Wilcoxon signed-rank tests were applied to assess differences between paired observations (e.g., baseline vs. final). Effect sizes and 95% confidence intervals were computed where appropriate to support clinical interpretability of statistically significant findings. For testing purposes, a *p*-value of 0.05 was considered statistically significant.

To identify subgroups of participants with distinct technological profiles, a k-prototypes clustering algorithm (*kproto*) was applied, which accommodates mixtures of continuous and categorical variables. Optimal number of clusters were identified via elbow method [24]. Solutions with k = 2 and k = 3 clusters were evaluated and compared. Cluster stability and separation were assessed using standard internal validation criteria. To characterize the relative contribution of each variable to cluster differentiation, three complementary approaches were employed: (i) Kruskal–Wallis tests for non-normally distributed variables, providing a non-parametric measure of between-cluster differences; (ii) inspection of cluster centroids to quantify each variable’s deviation from the overall mean; and (iii) loading analysis on the principal components most responsible for cluster separation, derived from Principal Component Analysis (PCA).

Correlation structure among digital adoption constructs was explored and visualized using *ggcorrplot*. All figures were produced using *ggplot2*.

Given the differences among pilot sites in participant characteristics, intervention duration, recruitment procedures and technologies, pilot site was considered an important contextual factor in the analysis. Longitudinal changes were first examined across the overall sample and were subsequently analyzed separately by pilot site. Socio-demographic and contextual variables, including age, educational attainment, and urban or rural residence, were also explored in relation to the outcomes.

## 3. Results

### 3.1. Pilot Sites, Study Duration, and Participant Enrollment

**Location:** The AGAPE pilot study took place in Italy, Portugal and Romania and was conducted by the research teams in Fondazione Don Gnocchi, Cáritas Diocesana de Coimbra (CDC) and Universitatea de Medicină și Farmacie Carol Davila (UMFCD), respectively.

The pilot implementation was adapted to the organizational context of each participating country while maintaining a comparable intervention framework. The study timeline spanned from February 2024 to January 2025. A description of the technologies delivered is provided in Table 1. 

In Italy, the duration of the pilot study was 9 months, from February 2024 to November 2024. The Italian AGAPE pilot was conducted in Marradi and Palazzuolo sul Senio. Participants followed a single pilot pathway structured around three assessment phases: baseline, intermediate and final. The study therefore covered most of 2024, allowing participants to experience the AGAPE technologies and coaching support across different seasons and daily life conditions.

In Romania, participant activities covered a 12-month implementation period (February 2024–February 2025). Participants were enrolled in three successive cohorts with different follow-up durations (4 months, 8 months, and 4 months), with the latter two cohorts partially overlapping. This recruitment strategy enabled evaluation of the intervention across different seasons and varying environmental conditions while ensuring continuous data collection throughout the study period.

In Portugal, the intervention was conducted in two sequential 4.5-month phases, together covering the planned 9-month study period. The first phase (March–July 2024) involved participants recruited from a parish council, a day care centre, and a community centre. The second phase (July–November 2024) was carried out in a day care centre using the same study protocol.

Participants were recruited from the beneficiaries of the healthcare/social care services the research organizations provide, as well as from the staff contact networks. Participants’ personal demographics and contact data were recorded and stored locally. At the initial meeting, after signing the informed consent, the participants received an individual code, including the acronym of the location, the stakeholder category and their own enrollment number. Further information on the participants’ characteristics is provided in Appendix B.

**Study activities:**

Participants received training and personalized coaching adapted to their level of digital adoption, combining digital literacy education, face-to-face and remote support, and technology-assisted feedback.

AGAPE coaches were selected based on their profession and assessed on key AGAPE topics (health prevention, active ageing, ICT/eHealth). Those needing further training attended sessions covering geronto-psychology, communication, digital health, smart environments, behavioural change, coaching methods, and related skills. Their background and personal traits were also considered important for building trust with older adults and caregivers.

Study participants were recruited and initial meetings were scheduled. In the initial meeting, older adults were thoroughly documented regarding the study, signed the informed consent and filled in the baseline the digital adoption questionnaire (DA) combined with a User Engagement Interview, followed by installation of the AGAPE Assistant app. Based on responses, a suitable device (wristband with visual interface, smart band for ankle or Smart Socks) was assigned and demonstrated, including key functionalities and Bluetooth connection to the app. The wearables were swapped between participants, after cleaning and disinfection, paired with their smartphone, as needed to avoid boredom, fit the adoption progress and maintain engagement. Additional questionnaires (SUS, technostress, EQ-5D-5L, UCLA) were administered. Participants were also asked to involve family caregivers.

IoChat was introduced and explained, as well, and installed on participants’ smartphones.

Formal caregivers, recruited by the research team, participated in individual interviews where informed consent was obtained and the AGAPE Monitor was presented. They were trained in its use and completed digital adoption, SUS, CarerQol-7D, and technostress questionnaires.

Group sessions were held for older adults and caregivers with low digital literacy to introduce active and healthy ageing concepts and the use of digital tools. Individual coaching plans were then developed and personalized for each participant, including goals, monitoring, and technology use adapted over time.

At mid-term, older adults attended a brief face-to-face evaluation including SUS and technostress questionnaires, qualitative feedback, and the option to switch devices.

At final assessment, all questionnaires were repeated and qualitative feedback on participation and coaching was collected. Devices were then returned and apps uninstalled.

**Participant documentation.** At enrolment, each participant received an individualized information package containing QR codes for accessing the AGAPE applications, together with detailed guidance on the study procedures, the digital tools, the AGAPE Monitor platform, the monitored parameters, and their baseline innovation adoption profile.

Throughout the intervention, participants were continuously supported by AGAPE coaches and healthcare or social care professionals, who provided education on digital literacy, eHealth, digital health technologies, AHA, and healthy lifestyle behaviours.

Older adults were equipped with the AGAPE digital ecosystem, including the AGAPE Assistant application installed on their Android smartphones and wearable devices selected according to the outcomes of the pre-validation phase. Available wearables included smart bands, sensor-enabled socks, and sensor straps. To encourage sustained engagement and familiarize users with different technologies, participants rotated between all wearable types during the study. Coaches selected and introduced the devices based on participants’ interests, progress, and individual support needs.

Informal caregivers, formal caregivers, coaches, and older adults all used the AGAPE Assistant application. Formal caregivers additionally evaluated the AGAPE Monitor platform, which enabled remote monitoring of participant progress. Coaches provided ongoing technical assistance, motivation, and personalized feedback to support continued technology use and engagement with digital health and active ageing concepts. Consequently, usability scores obtained with the SUS reflected participants’ experience with the integrated AGAPE ecosystem rather than a single device.

### 3.2. Study Population

The total study sample comprised 217 participants recruited across the three pilot sites in Italy, Romania, and Portugal. The final sample, after the removal of drop-outs and incomplete cases, included 146 OAs, 24 informal caregivers (ICs), and 48 formal caregivers (FCs). Among the OAs, 49 were recruited in Italy, 49 in Romania, and 48 in Portugal. Overall, OAs had a mean age ranging between 69 and 72 years, with a predominance of females (68%) and a high proportion of retired participants. The ICs and FCs groups showed an even stronger female predominance (79%) and were generally younger and more highly educated than the OAs. ICs were primarily family members and often lived with the OAs, whereas FCs were all employed and predominantly based in urban environments.

Following data cleaning, the present analysis focuses on the OAs sample. Among the 146 OAs, approximately 68% were female, 28% lived alone, and 37% lived in a rural environment. 34% of OAs had a low level of education. The distribution of OAs across the three pilot sites was highly balanced, whereas the proportion of Carers was considerably higher in Portugal (46%) than in Italy and Romania. Further differences emerged in relation to the living environment and educational attainment of the OAs. The Italian sample was predominantly recruited from rural areas (93%), in contrast to the Romanian sample, in which only 6% of participants lived in rural environments. The Italian OAs also generally had lower educational attainment than participants recruited in Romania and Portugal. Conversely, a higher degree of balance across pilot sites was observed for other demographic characteristics, including gender and marital status. All OAs lived in their own homes; therefore, the “living conditions” variable was excluded from subsequent analyses. In addition, some questionnaires were excluded because of inconsistencies in completion times or duplicated responses. Table 2 presents the number of participants by pilot site, together with the devices delivered for use during the pilot study.

For the technostress analysis, the analysis-specific sample comprised 75 OAs, of whom 64% were female, 64% were over 70 years of age, and 33% lived alone. Participants were distributed across Italy (63%), Portugal (14%), and Romania (24%). A total of 75 baseline assessments were available, together with 68 intermediate and 55 final assessments.

For the DA analysis, the analysis-specific sample comprised 100 OAs, with 67% female participants, 70% aged over 70 years, and 33% living alone. Participants were distributed across Italy (47%), Portugal (35%), and Romania (18%). In this sample, 100 baseline assessments and 72 final assessments were available.

Finally, the SUS analysis included 74 OAs, of whom 64% were female, 64% were over 70 years of age, and 32% lived alone. The sample included participants from Italy (64%), Portugal (12%), and Romania (24%). A total of 74 baseline assessments were collected, together with 67 intermediate and 55 final assessments.

### 3.3. Quantitative Results

Because the three pilot sites differed in intervention duration, recruitment setting, participant profile, and technology package, the longitudinal outcomes were interpreted both at the overall sample level and separately by pilot site. Overall estimates describe the direction of change across the multinational implementation, whereas site-stratified findings were used to examine heterogeneity related to local context and implementation characteristics. To ensure the validity of longitudinal comparisons, survey responses were pre-processed according to the elapsed time between assessment points. Some questionnaires were excluded due to inconsistencies in timing of completion or duplication of responses.

Only records with a minimum interval of 135 days (4.5 months) between baseline and final assessments, and of 60 days (2 months) between baseline and intermediate assessments, were retained. These temporal thresholds were defined a priori at project level, balancing the need to ensure a meaningful exposure period to AGAPE services with the requirement to preserve an adequate and statistically meaningful sample size across all three pilot sites. This quality-control procedure was applied at questionnaire record level and resulted in the exclusion of records that did not meet the required timing criteria: 8 EuroQoL, 10 UCLA, 4 CarerQoL, 184 SUS, and 42 technostress questionnaire records.

**Figure 1:**
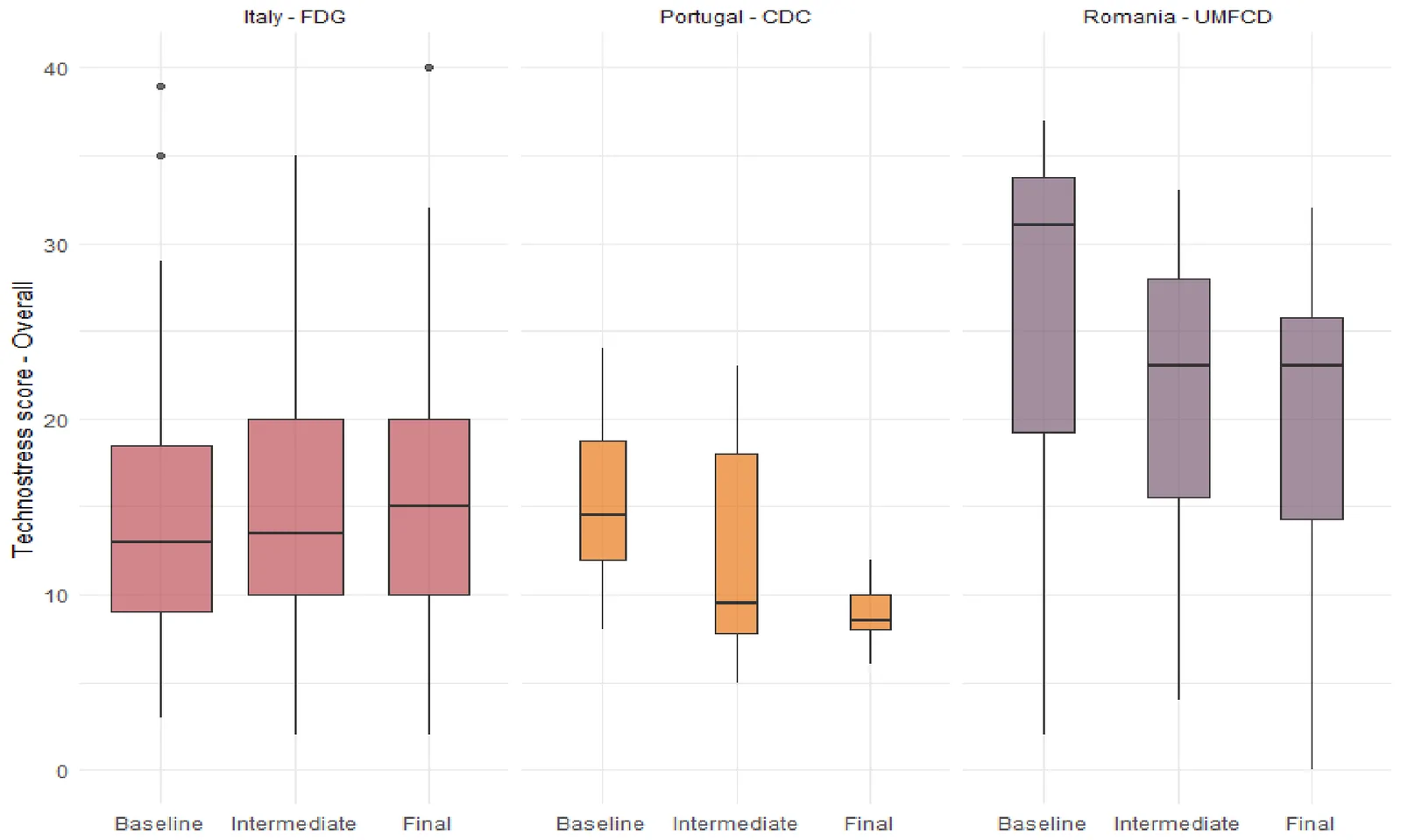
Boxplot of technostress overall score by phase and pilot site.

To investigate whether prolonged exposure to the digital platform influenced technology-related stress among older adults, longitudinal changes in technostress scores were analyzed across assessment time points and pilot sites. Technostress analyses were conducted on 75 older adults aged over 65 years. Overall technostress scores showed a decreasing trend from baseline to final assessment, although the reduction did not reach statistical significance at population level (Wilcoxon test, *p* > 0.1). Significant differences emerged across pilot sites (Figure 1). Romanian participants reported substantially higher baseline technostress levels than Italian participants (+11.4 points, *p* < 0.001), but also experienced the largest improvements over time. Significant reductions were observed in the Portuguese (CDC) cohort at the final assessment (−6.34 points, *p* < 0.05) and in the Romanian (UMFCD) cohort at both intermediate (−4.50 points) and final assessments (−6.39 points; *p* < 0.05). To explore whether contextual factors influenced the intervention outcomes, technostress scores were also compared according to participants’ living environment (urban versus rural). Living environment was associated with technostress: participants residing in rural areas reported lower baseline scores than urban participants (−8.15 points, *p* < 0.001), although this difference was no longer evident at final assessment, suggesting a relative increase in technostress among rural users over time. Analysis of the individual dimensions revealed that reductions in CDC scores were mainly driven by decreases in techno-overload (−2.28 points, *p* < 0.05) and techno-invasion (−2.20 points, *p* < 0.05), whereas UMFCD participants showed significant decreases in perceived complexity (−2.06 at intermediate and −3.01 at final, *p* < 0.05) and techno-invasion (−1.50 at intermediate and −1.89 at final, *p* < 0.01).

**Figure 2:**
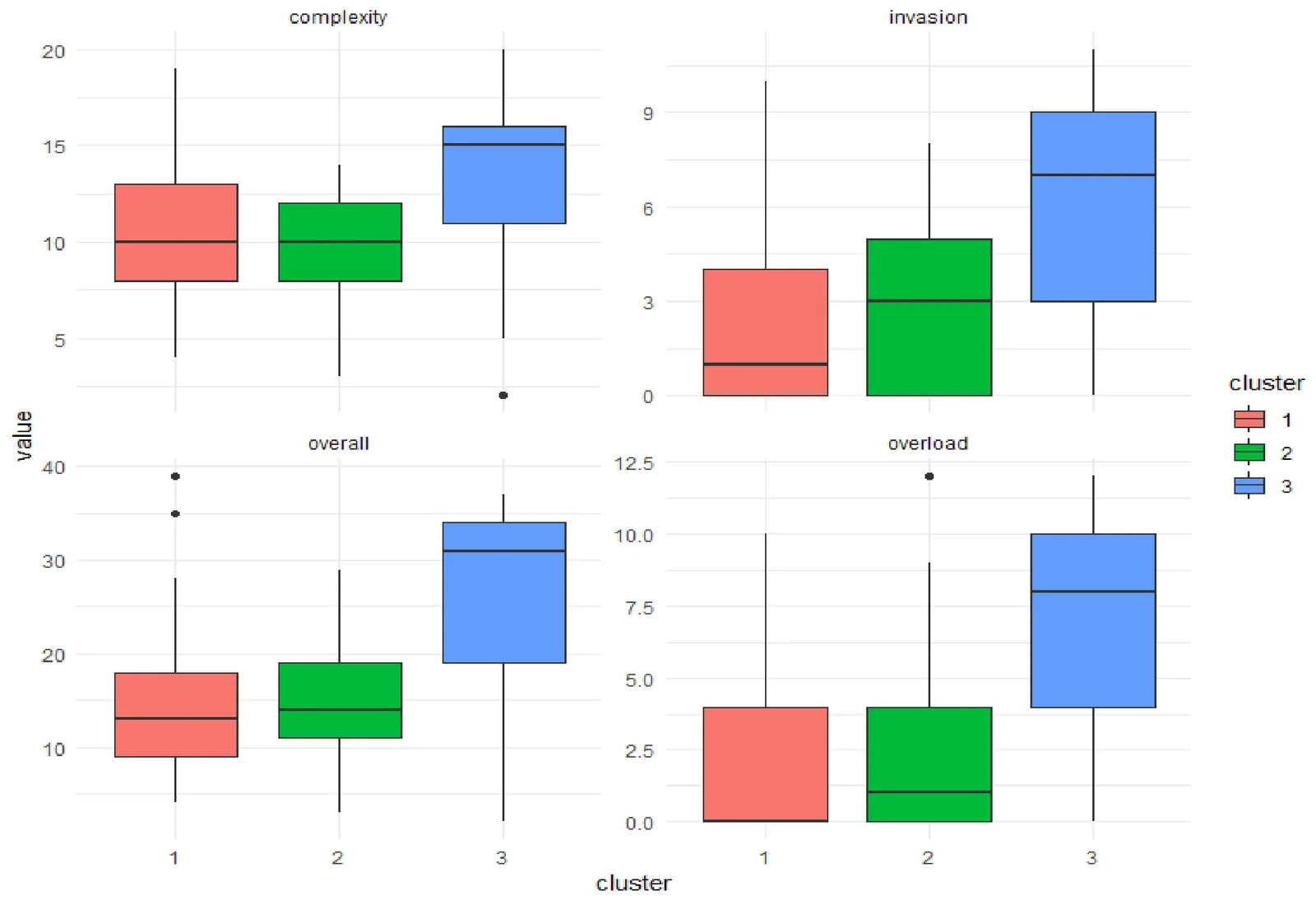
Boxplot of technostress overall score and subscores by identified k-prototype clusters.

To further investigate heterogeneity in technostress, a k-prototypes clustering analysis based on socio-demographic variables identified three distinct groups (Figure 2):

- The first cluster (median = 13.0; mean = 14.92) consisted predominantly of participants over 70 years of age, with low educational attainment and living in rural areas, mainly in the Italian Mugello region, and exhibited the lowest technostress levels.
- The second cluster (median = 14.0; mean = 14.94) showed comparable overall technostress scores but slightly higher techno-invasion, and included somewhat younger and more educated participants, incorporating all Portuguese participants.
- The third cluster (median = 31.0; mean = 26.53) displayed substantially higher technostress levels across all dimensions and was composed entirely of urban Romanian participants, who were generally younger and more highly educated.

**Figure 3:**
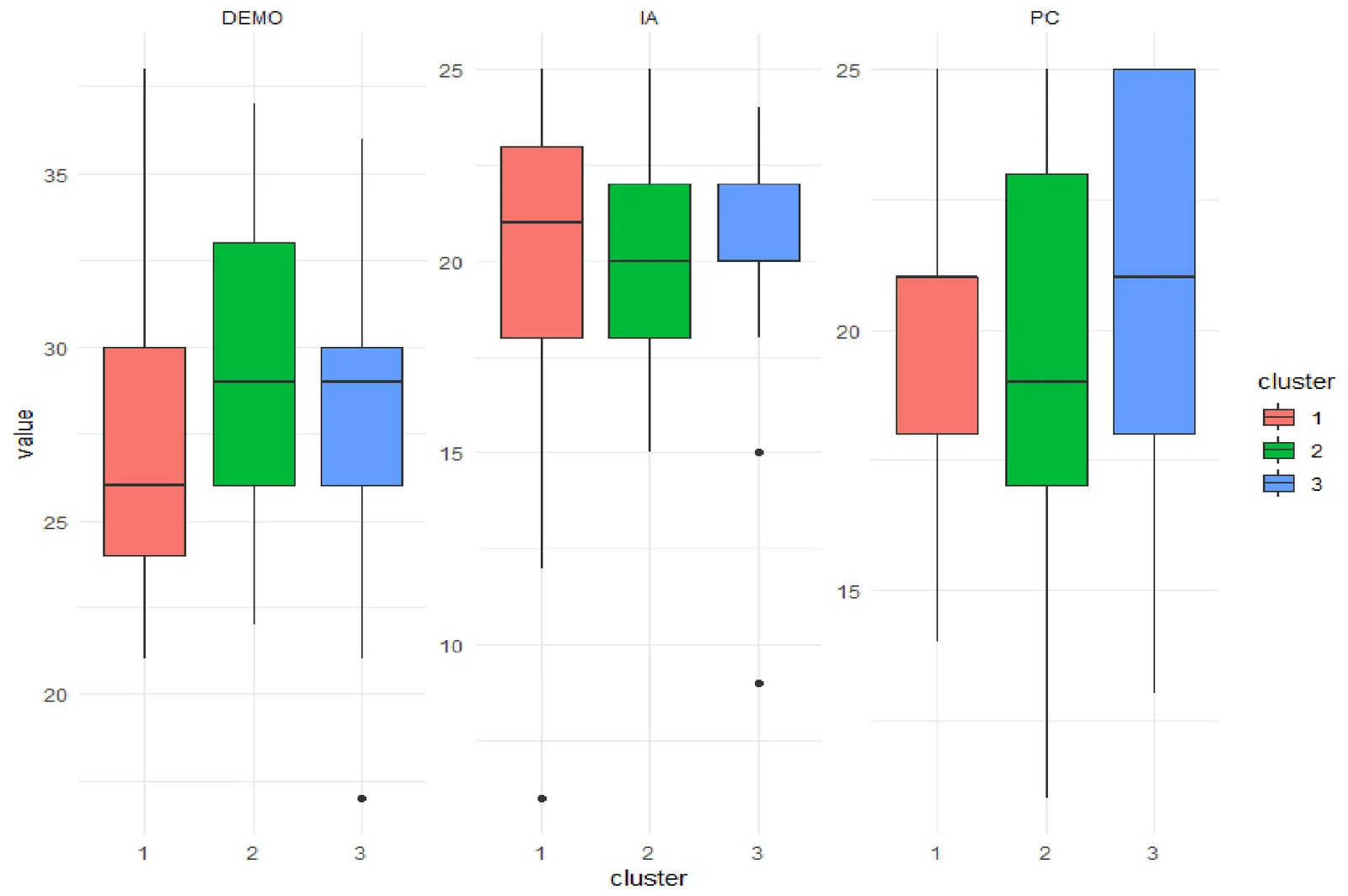
Boxplot of digital adoption emphasizing a series of important factors (intention to adopt—IA; privacy concerns—PC; and previous experience/comfort/familiarity—DEMO) by identified k-prototype clusters.

To investigate determinants of digital technology acceptance and their evolution during the intervention, DA scores were analyzed longitudinally and according to socio-demographic characteristics. When DA variables were included in the clustering procedure, considering the three most influential dimensions identified during feature selection (intention to adopt, privacy concerns, and previous experience/comfort/familiarity with technology), Cluster 1 remained characterized by the lowest familiarity with technology, whereas Cluster 3 combined lower privacy concerns with a stronger intention to adopt digital solutions and greater technological familiarity. Cluster 2 showed intermediate characteristics, resembling Cluster 1 in terms of privacy concerns and intention to adopt, while being closer to Cluster 3 with respect to technological familiarity (Figure 3).

**Figure 4:**
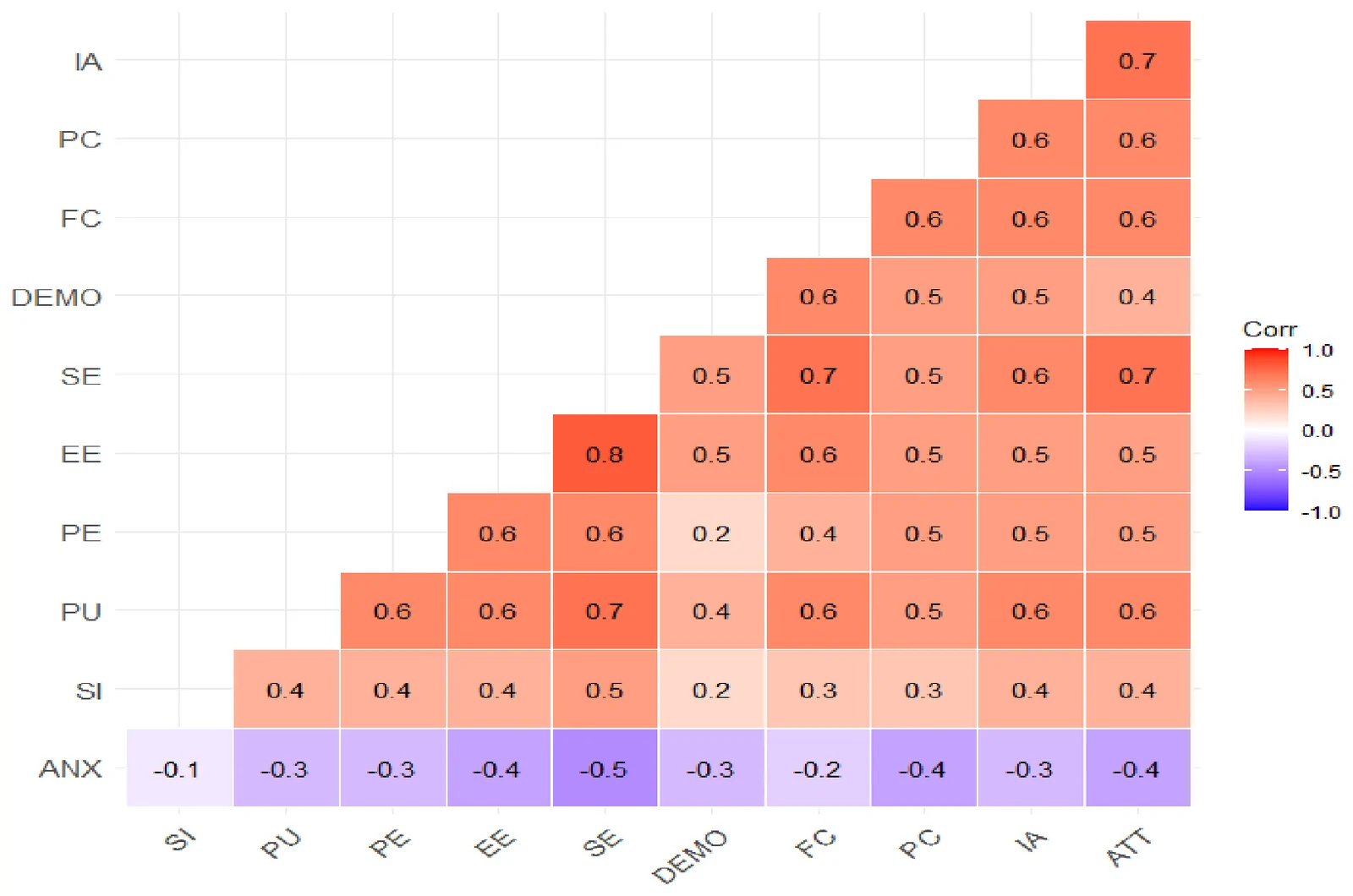
Correlation plot of digital adoption questionnaire’s items. Legend: attitude toward technology (ATT): overall disposition and feelings toward technology usage; self-efficacy (SE): confidence in one’s ability to successfully use technology; facilitating conditions (FC): perception of the availability of resources and support to use technology effectively; performance expectancy (PE): expectation of the benefits and effectiveness of using technology; effort expectancy (EE): perception of the ease of using technology; privacy concerns (PC): concerns about the privacy and security implications of using technology; social influence (SI): influence of social factors on technology adoption decisions; anxiety (ANX): feelings of unease or discomfort associated with using technology; demographics (DEMO): assess the overall familiarity and voluntariness in using any common digital tools (smartphone, tablet, laptop, availability of internet connection); perceived ubiquity (PU): perception of the availability and prevalence of technology in daily life; intention to adopt (IA): willingness and readiness to adopt and use technology.

The DA questionnaire was administered to 100 older adults aged over 65 years. The instrument was developed through a structured adaptation of the Unified Theory of Acceptance and Use of Technology (UTAUT) framework and integrated additional determinants of digital health adoption identified in the recent literature. Correlation analysis of questionnaire items confirmed meaningful relationships among the main theoretical constructs, including effort expectancy, performance expectancy, social influence, perceived ubiquity, self-efficacy, privacy concerns, intention to adopt, anxiety, facilitating conditions, attitude toward technology, and familiarity with digital tools (Figure 4).

To explore the determinants of digital technology adoption, an ad hoc digital adoption questionnaire based on UTAUT [18] was administered to 100 adults aged 65 years and older. The questionnaire was expanded to incorporate additional factors associated with digital health adoption factors identified in the recent literature, providing a broader assessment of technology acceptance in older populations.

Correlation analyses show significant associations among the principal constructs included in the instrument (Figure 4). These comprised performance expectancy, effort expectancy, social influence, facilitating conditions, self-efficacy, perceived ubiquity, privacy concerns, anxiety, attitude toward technology, intention to adopt, and familiarity with digital technologies, supporting the theoretical structure of the questionnaire.

**Figure 5:**
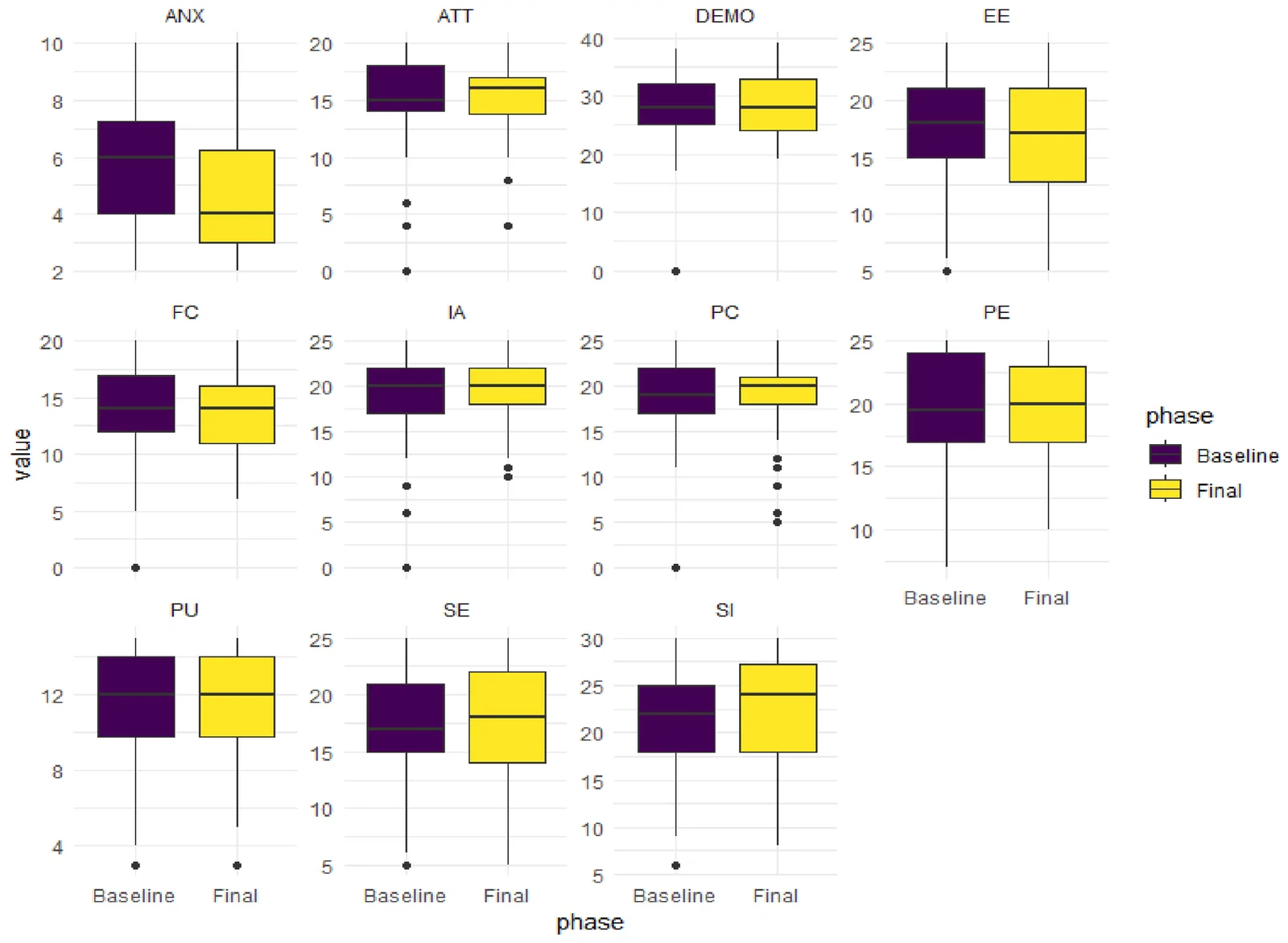
Boxplot of digital adoption questionnaire’s items scores by phase.

After data cleaning, including the removal of incomplete responses, duplicates, and assessments with insufficient longitudinal intervals, 72 matched baseline–final records were retained for analysis (Figure 5). The sample consisted predominantly of older adults, with only seven informal carers who tested intensively the platform, under the same study design as the older adult participants; 18 participants were recruited in Romania, while Italy and Portugal each contributed 27 participants.

Additional analyses revealed a significant decrease in technology-related anxiety between baseline and final assessments (Wilcoxon test, *p* < 0.05) and a concurrent increase in the influence of relatives, healthcare professionals, and peers on technology adoption decisions (*p* < 0.05). Educational attainment emerged as the socio-demographic factor most strongly associated with digital adoption outcomes, showing positive associations with attitude toward technology, effort expectancy, facilitating conditions, intention to adopt, and self-efficacy. Age was negatively associated with familiarity with digital technologies, whereas living environment was associated with both perceived ubiquity and social influence, with rural participants reporting increased perceptions of technology availability and reduced reliance on social influence over time.

**Figure 6:**
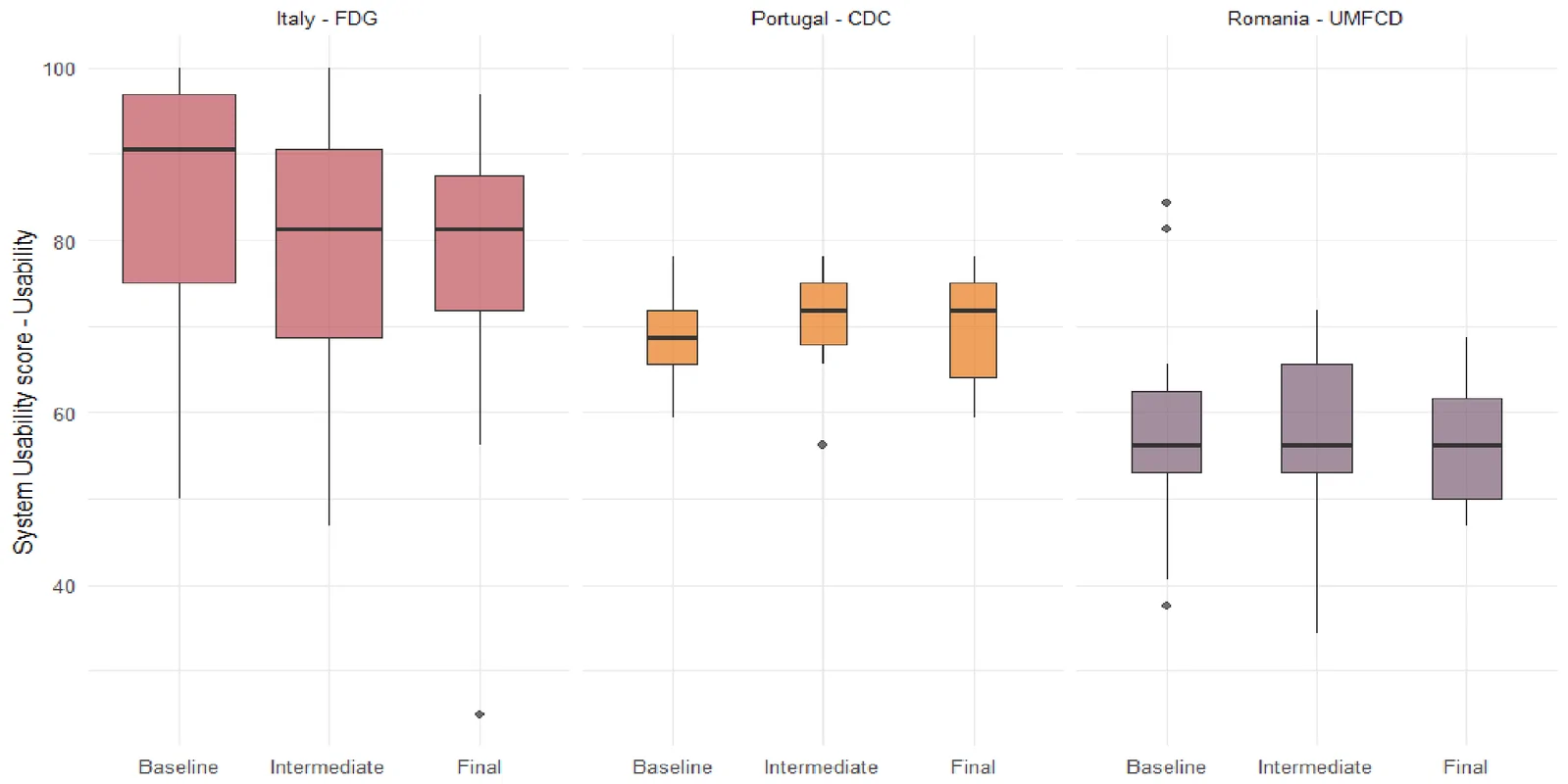
Boxplot of SUS usability score by phase and pilot site.

**Figure 7:**
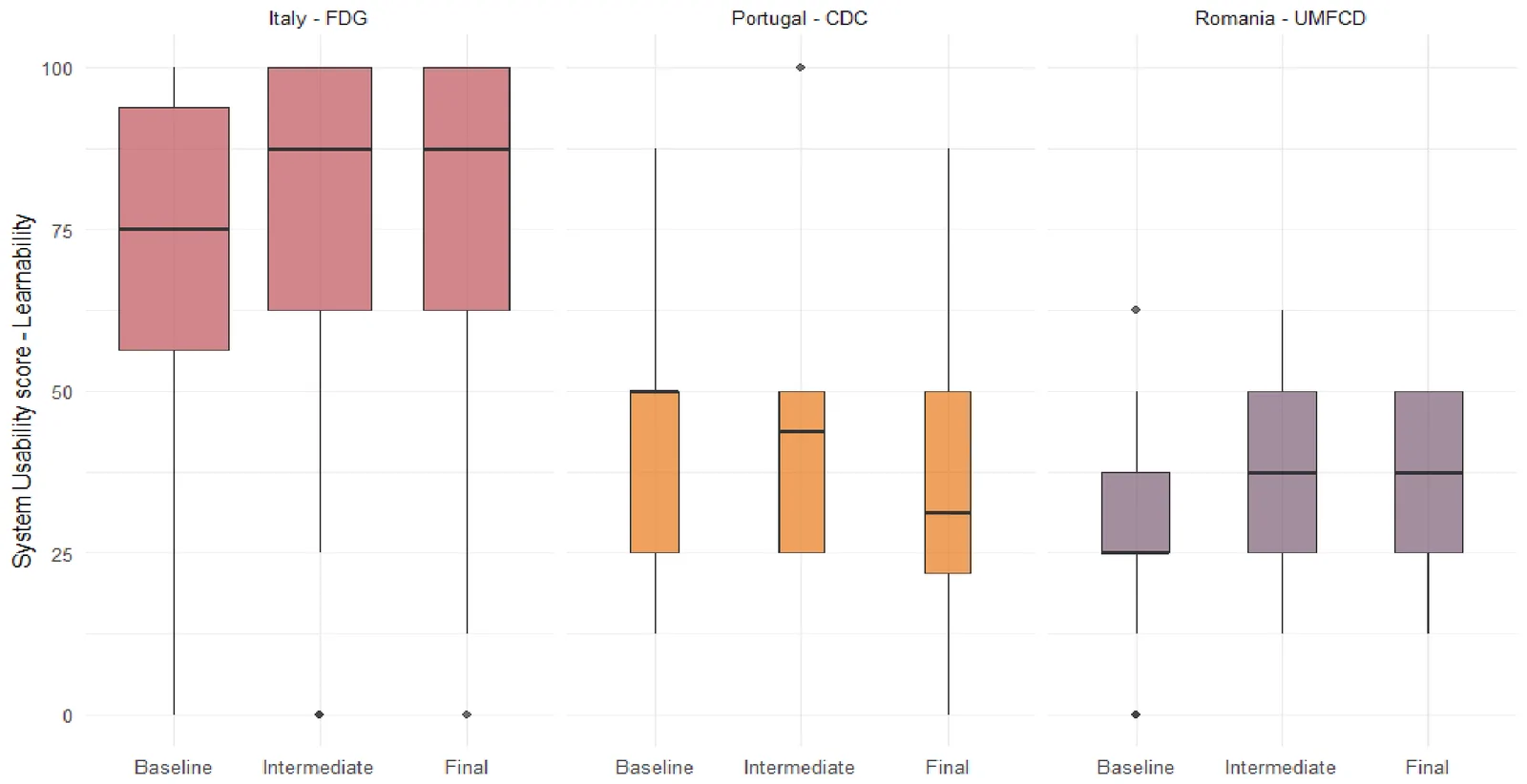
Boxplot of SUS learnability score by phase and pilot site.

**Figure 8:**
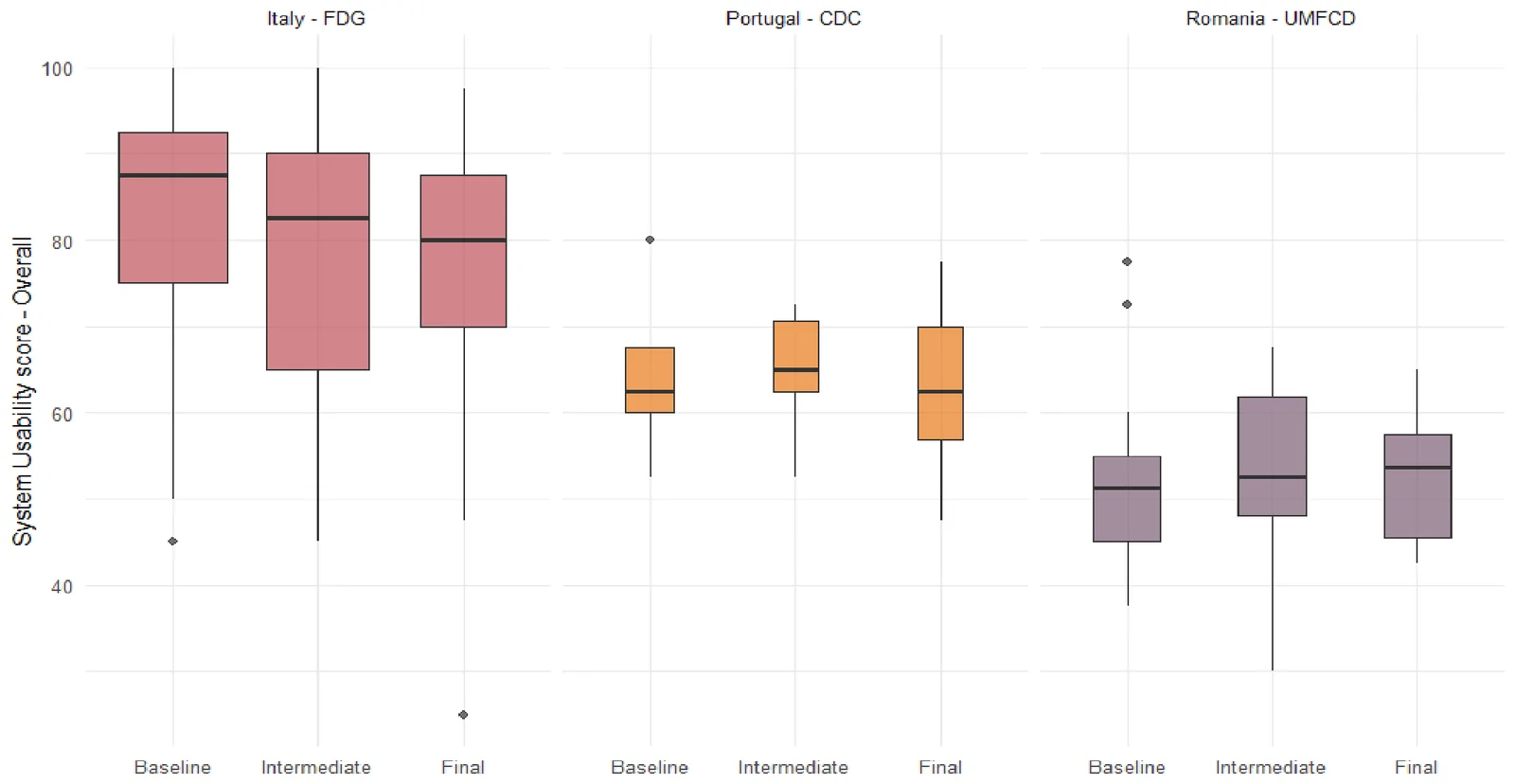
Boxplot of SUS overall score by phase and pilot site.

To evaluate participants’ perception regarding the usability and learnability of the digital platform, longitudinal changes in SUS scores were examined overall and across pilot sites. Usability analyses were conducted on 74 older adults using the SUS, which was decomposed into usability and learnability subscales (Figure 6, Figure 7 and Figure 8). Overall SUS scores significantly decreased over time (Wilcoxon test, *p* < 0.01), whereas Learnability scores remained stable. Marked differences were observed across pilot sites. At baseline, participants from CDC and UMFCD reported significantly lower usability and learnability scores than those from the Italian site, with average differences ranging from −17.6 to −27.9 points for usability and from −29.5 to −46.2 points for learnability. The overall decline in SUS scores was largely driven by the Italian cohort, which showed significant reductions in both overall SUS scores at final assessment (−7.9 points; 95% CI: −13.04 to −2.79) and in the Usability subscale at intermediate (−6.5 points; 95% CI: −11.2 to −1.8) and final assessments (−9.6 points; 95% CI: −14.9 to −4.3). No significant longitudinal changes were observed in CDC, whereas UMFCD participants exhibited a significant increase in Learnability at final assessment (+9.03 points; 95% CI: 0.11–17.95). Educational level and living environment were also associated with usability outcomes, with higher educational attainment and urban residence corresponding to better usability and learnability scores, consistent with the demographic profile of the UMFCD cohort.

### 3.4. Heuristic Evaluation and Descriptive Participant Feedback

The findings presented below represent a descriptive synthesis of the structured heuristic assessments and participant feedback. They should not be interpreted as results of a formal qualitative or thematic analysis.

The overall heuristic evaluation indicated a generally positive perception among formal caregivers, older adults, and family members regarding the solution tested during the pilot, with consistent ratings across groups. The system was appreciated for its alignment with real-world expectations, consistency, minimalist design and reliance on recognition rather than recall. The user manual and the coach’s role in supporting error recovery were also considered strong positive elements.

However, some limitations were identified, particularly regarding user control, visibility of the system status and error prevention, which were rated more neutrally and showcased as requiring improvement. Technical issues including sensor connectivity problems and occasional app crashes reduced overall perceived efficiency of use. Flexibility was intentionally limited to support simplicity of interfacing to ease technology use for the older adults, an aspect considered appropriate by participants. The absence of full in-app guidance was noted but largely mitigated by the presence of the coach and by the detailed paper-based user manual.

From a usability perspective, the solution was seen as supporting learning-by-doing, providing cognitive support (for memory and executive functions), and presenting high accessibility, including features for visually impaired users. would have appreciated to have access to more integrated parameters within the app. Despite neutral ratings for overall user experience and navigation fluidity, the system was not perceived negatively in this regard, though.

UTAUT-related findings: participants reported that the solution partially met their needs, increased motivation to use digital tools, improved perceived safety and supported healthier lifestyles without bringing stigma or dependency concerns. External support from the medical teams and family caregivers reinforced technology acceptance. The coach was considered essential in compensating technological limitations and ensuring continuous motivation and engagement.

Multiple tools were tested, participants primarily adopting the wearable devices combined with the AGAPE Assistant app. The smart band was the most appreciated wearable due to its familiarity and range of physiological measurements, while Smart Socks were valued mainly for clinical insights but considered less practical for home use. The AGAPE Monitor received highly positive evaluations from coaches, particularly for system status visibility, navigation, data richness, consistency, and design quality. It was also perceived as promoting learning-by-doing, simplifying information processing, supporting recognition-based interaction and enhancing motivation and social participation, resulting in an overall excellent user experience rating from coaches and the other formal caregivers as well.

## 4. Discussion

The population is ageing worldwide, due to the latest improvements in life conditions and to progress in healthcare. Population ageing comes with challenges due to the increase in numbers and complexity of chronic health conditions and to the associated disabilities. The community as well as for the healthcare systems are highly burdened as a consequence [25]. This situation is associated with the lack of specialized staff for long-term home or institutionalized care and with the reduced QoL of older people with disabilities.

The AGAPE project involved three core user groups: older adults (primary users), care professionals and family caregivers (secondary users), community and organizations (tertiary users). By directly engaging all stakeholder categories in the design and implementation process, the project ensured that the services provided to the beneficiaries reflect genuine needs, cultural contexts, and levels of digital readiness, responding to many questions expressed also in the literature [26]. This participatory approach fostered trust and ensured participants’ adherence to the study and the long-term commitment to study activities. It also allowed testing the AGAPE services with all potential beneficiary categories.

Gerontotechnologies developed lately, including in AAL Projects, have the potential to enable and support ageing in place by fostering functional independence, activity and social reconnection, with subsequent benefits in terms of quality of life and healthy ageing [27]. As a counterweight, low digital literacy, high technostress and digital anxiety reduce older adults’ level of engagement with and the adoption of digital technologies [28]. Costs and lack of awareness regarding availability, user friendliness and benefits of dedicated digital health pose relevant barriers in older people’s attitude and active behaviour towards digital technologies, deepening the digital gaps between generations [8]. The fact that informal family caregivers take upon themselves the tasks requiring digital knowledge and skills provide easy and safe solutions for the tasks, but hinder even more the possibility to improve in digital literacy of the older adults [29].

Ageing is also frequently accompanied by sensory, motor, and cognitive changes that require digital technologies to incorporate age-appropriate design features and accessible functionalities [30,31]. Older adults also tend to benefit more from observational learning, individualized face-to-face instruction and hands-on practice than from abstract or written instructions alone [32,33]. This supports the potential relevance of human coaching within multicomponent digital health interventions for older adults.

Not all user-interface recommendations taking into account perception, cognitive and physical ability barriers of older adults are implemented in the technologies developed for older adults yet, adding new obstacles to digital adoption [31,34]. Personalized training and coaching may improve perceived usability and self-efficiency in relation to digital technologies, bringing also benefits in social engagement and lifestyle [35].

Coaching older adults has relevant benefits in regards of well-being, social engagement, active reconnection with family and peers and improved lifestyle with healthier behaviours [36]. While coaching for ageing well through digital means proves beneficial for younger older adults transitioning from work life to retirement [36].

Digital technologies that enable older adults to monitor physical activity and vital health parameters can encourage their involvement in self-management and support independent living. When users recognize these technologies as beneficial for addressing their health needs, they are more likely to adopt them. According to technology acceptance models, this behaviour is primarily influenced by perceived usefulness, together with perceived ease of use and the influence of the user’s social environment [37]. AGAPE solution proposed user-friendly technologies, with dedicated training and support, provided to users in personalized manner, but asking user to manage aspects like charging devices, connecting wearables to the AGAPE Assistant app, accessing different functions of the technologies to visualize parameters, learn to use a new communication app, to populate a profile and manage contacts and communication. The AGAPE coach provided assistance as needed, the aim being to enhance self-efficacy of the older adult and solution finding behaviours, before them asking for assistance. As part of the multicomponent AGAPE intervention, key to the coach’s work was the ability to establish an empathic and trust-based relationship with the older adults, adapting the support they offer to the individual needs, digital skills, health literacy and personal circumstances of the older participants [11]. This approach based on supervised tasks and challenge-driven exploration improved the attitude of older adults towards technologies, in terms of reducing the level of the technostress along the study. A thorough analysis of the technostress determinants would also ask for taking into account cultural specificities [38]. The active involvement of family caregivers who support OAs included in the study to learn and engage, enhanced even more the motivation of the older adults, as described also in other recent studies [39].

The progress of the usability scores indicates higher initial scores on SUS, probably due to the initial intense support of the coach and to high expectations of participants. The intermediate evaluation shows lower scores probably due to the challenges raised by the daily use of the devices, while the final evaluation shows again an increase in SUS scores, probably following the habituation of the users with all the tasks and specificities of the devices, as stated by participants themselves.

SUS is used in studies analyzing the engagement of older adults with technologies, on a usual basis, being recognized as a reliable and valid assessment tool for this population [40]. The results of the present study show a significant decline in perceived usability over time (Wilcoxon *p* < 0.001), consistent across all three pilot sites. This is in accord with the digital literacy and skills in the population aged 65–74 years old in Europe—only 25% of the European population in this age category has basic digital knowledge and skills. Statistics do not take into account people over 75 years old [40]. Romanian users reported consistently lower usability scores compared to participants in Italy and Portugal throughout the study. This is explainable by the fact that while in 2023, 55% of the population 15–74 years old in Europe had at least basic digital skills, only 28% of the Romanian population in the same age group could say the same. The level of digital literacy is highly influenced by the level of formal education, also [41,42]. SUS scores reflect previous experience as well as user friendliness of technology and user’s expectations. AGAPE technologies showed usability scores decreasing over time, suggesting possible issues related to interface complexity, technology fatigue or growing expectations as users became more familiar with the system, or a combination of those. A 2026 meta-analysis of 105 studies found a strong correlation between SUS and perceived workload, as well as weak correlations between SUS, task time and error rate [43]. The learnability subscore did not change over time in the AGAPE pilot. The improvement in SUS scores observed toward the end of the study may reflect, at least in part, participants’ increasing familiarity with the technologies and development of digital skills over time. Previous evidence suggests that continued technology use and habituation may contribute to greater efficiency, reduced cognitive load and support needs, and improved interaction flow [44,45,46].

The previous statement is supported also by the progress of the technostress scores. Technostress scores decreased between the three assessment points in all sites, in parallel with reduction in perceived overload, complexity and technology invasiveness, as participants got used with the wearables, the apps, actions needed to ensure connectivity, functionalities, and solutions for different issues under the close monitoring and coaching of the AGAPE coaches. Our study suggests that training, gradual adoption of technologies, peer support and hands-on experience, along with personalized coaching, have the potential to improve adoption while reducing technostress levels. Similar results were obtained in a 2025 study regarding the technostress level among older workers as well as in other previous works [47]. Anxiety and living alone are associated with lower acceptability of new technologies, while exposure time and social support reduce the technostress and improve the acceptance of digital technologies in older adults [46,48,49]. The AGAPE study encouraged more intensive interactions between older adults and their family members, while also generating partnerships between the participants and the coaches. Health and lifestyle coaching, including using digital technologies, is very well received by older adults, even more than by their younger community members [47].

The results regarding technostress and usability levels are in accord with the progress of digital adoption questionnaire aspects related to effort expectancy, fear of making mistakes, self-efficacy, facilitating conditions. Social influence, the level of formal education and previous experience have a positive influence on the digital adoption level, while high expectancies, low self-efficacy and high techno-anxiety are associated with lower levels of digital adoption. Digital literacy and skills improvement in old age requires specific strategies. The beneficiaries must undergo a perception shift for which they need emotional reinforcement and supportive social environments to boost their interest and motivation for acquiring new skills enabling social participation as well as independent living [49]. Ageism and self-perceived low efficiency deeply affect the psychological determinants of digital adoption, with negative health-related outcomes and negative trends of social engagement [48,49]. The digital adoption questionnaire findings demonstrated potential benefits of human coaching to help OAs to remain engaged in technologies and improve skills over time. Longitudinal analysis of 72 matched baseline–final records shows that most participants initially belonged to mid-level digital adoption profiles, reflecting mild-to-moderate digital skills. Although profile transitions were not always linear, findings suggested an overall improvement in users’ confidence and familiarity with technology, particularly in perceived digital competence and self-efficacy. Predictive modelling identified technology acceptance, effort expectancy, self-efficacy and digital confidence as the main behavioural domains associated with digital adoption outcomes. Additional results highlighted a significant reduction in anxiety and hesitation toward engagement with technology, alongside a growing influence of the social support networks in motivating technology adoption. Socio-demographic analyses further showed that the level of formal education positively influenced most digital adoption dimensions. Overall, the results support the relevance of personalized coaching and adaptive digital literacy strategies in fostering sustainable engagement with active and healthy ageing technologies.

The analysis of the digital adoption questionnaire highlighted the critical role of specific behavioural domains—particularly effort expectancy and self-efficacy—in influencing the adoption of technology among older adults. These domains emerged as strong predictors of users’ final innovation adoption profiles, underscoring their relevance in shaping digital engagement outcomes. While the overall progression across predefined adoption profiles (from lowest A1 to the highest B2) was not uniformly conclusive—potentially due to refinements in the classification algorithm during the pilot, which may have altered criteria between baseline and final assessments—the findings nonetheless point to a clear and significant improvement in participants’ digital skills. In some participants, the DA profile decreased in rank, as a consequence of the improvement of the awareness level regarding their own knowledge and skills. This positive trend suggests the potential of AGAPE’s tailored coaching strategies and service design in fostering digital readiness.

Finally, the variability in user progression highlights the complexity of digital behaviour and the need for continuous personalization and dynamically tailored support strategies. The methodology applied in this analysis also underscores the importance of going beyond simple metrics to understand user transformation in long-term digital health interventions.

### 4.1. Local Specificities

The three pilot sites presented with distinct demographic and geographical contexts, each facing specific challenges related to ageing, access to care and social participation.

Romania: The Romanian pilot was implemented in Bucharest, where adults aged 65–69 years represent the largest proportion of the older population, with women outnumbering men. Most participants (95%) lived independently and owned their homes, reflecting the high homeownership rate reported nationally (94%) [50]. Despite living in an urban environment, older adults frequently experience social isolation. According to the Never Alone (Niciodată Singuri) survey, around one-quarter of adults aged 65 years and older reported feeling lonely very often, approximately 60% mainly engaged in household activities, and nearly one-third lacked someone to rely on or socialize with when needed [51]. A third part of the older people have no one to socialize with or rely on in times of need [51]. Access to services is low already due to the socioeconomic challenges and is associated with the capability to manage online and in person information gathering and visits to organizations.

Italy: The Italian pilot was conducted in the rural municipalities of Marradi and Palazzuolo sul Senio, located approximately 35 km from the nearest urban centres. The predominantly older population faces limited access to healthcare and other essential services because of geographical isolation and inadequate transport connections. These challenges became even more pronounced following the landslides that affected the area in 2023.

Portugal: The Portuguese pilot took place in Coimbra, a city in central Portugal where more than one-quarter of residents were aged over 64 years according to the 2021 Census. Similar to national demographic trends, population ageing is associated with increased risks of loneliness, difficulties in accessing services, and a growing need for continuous support to maintain health, independence, and quality of life.

More specific information can be found in the section Appendix B for the three countries involved in the AGAPE project.

In Italy, in summary, the value generated by AGAPE materialized in improved social inclusion, improved access to digital health, technological education, and continuous support enabling older adults to live healthier, more connected and more independent. In Portugal, the AGAPE ecosystem improved access to digital health, digital literacy and social inclusion. Tools like IoChat and Seniorphone reduced isolation, while the inclusive design ensured accessibility for all. The AGAPE coach played a key role in personalizing services and guiding users in adopting technology based on their individual needs. In Romania, the exploration of the personalized coaching and the continuous adaptive support of the professionals and of the family caregivers contributed to the improvement of the social and personal life experience of all actors in AGAPE pilot and provided intensive cognitive stimulation for participants. The impact of this stimulation was seen especially in the individuals with the higher risk of frailty, showing improvements in activity areas like playing serious games, maintaining focused attention on news and remembering events and appointments. The research highlighted an alarming situation regarding the low degree of social participation of older adults, as well as of the family and professional caregivers living in a big city like Bucharest. We mention the fact that in the UMFCD site, the research team explored the application of Functional Activities Scale (FAS)—assessment tool evaluating the older adults’ ability to perform instrumental activities of daily living. The scale is designed to help in the inclusion of subjects in the categories of people with normal cognition, mild cognitive impairment or mild dementia [52], and indicates the degree of dependency in regards of ADLs and iADLs of the older adults. Sum score range is between 0 (normal functioning, no activity limitation) and −30 (total dependency, physical and cognitive major deficits). Cut-point of 9 (dependent in three or more activities) indicates impaired function and possible cognitive impairment [52]. Five OAs presented difficulties in more than three domains (scores of 4–11), indicating the presence of mild cognitive impairment (already acknowledged at the inclusion moment just for three of them), and physical functioning deficits (motor and sensory deficits). No changes were noticed in FAS scores during the pre-pilot, but during the pilot, changes were obtained in one or two items, with a maximal change of two points (one per each item) in two items at the same person. Initial FAS scores included an average score of 1551. A total of 35 people presented with FAS score 0 and maintained that same score for the final assessment.

Improvements were seen in nine participants (one item with one point in eight persons, two items with one point each in two participants with medium FAS scores initial—7 and 4 respectively). Thus, main changes were seen in: item 4 (hobbies, games)—five participants improved in this respect; item 3 (shopping alone)—two participants; item 9 (remembering appointments)—two participants; item 5 (iADL with home appliances); item 7 (keeping track of events); item 8 (discussing news, TV); item 10 (travel arrangements and performance)—one participant each. This effect is most probably a result of the combination of training new practical skills along with the increase in social engagement with the coaches and peers. The changes were not associated with the duration of the participation (4 months versus 8 months). No changes were seen in FAS scores for the people with high independent functioning.

Despite common barriers, including limited digital skills and digital self-efficacy, the AGAPE intervention combined professional support with personalized training to facilitate participants’ engagement with digital technologies and behavioural adaptation. The findings suggest that the AGAPE model has the potential to provide a scalable and inclusive digital ecosystem integrating health and social care services while supporting older adults’ autonomy, well-being, and dignity.

The use of AGAPE Monitor allowed professionals to explore telecare potentialities of AGAPE platform and to generate awareness and interest of local tertiary user representatives in regards of the benefits of AGAPE (and generally remote monitoring) can bring as support for telerehabilitation services, allowing home and objectively-based disability prevention interventions as well as the continuity of care which is mandatory in order to ensure the success of preventative and rehabilitation programmes.

During the study, the identified facilitators of the user experience and adherence include: the **personalized coaching** seems to have relevance for adoption, usability support, and digital empowerment; the **remote monitoring** and the **quantified feedback** regarding vital parameters probably increased motivation and engagement; **peer interaction** opportunities which boosted the sense of belonging and motivation to go forth. Some apparent *barriers* to the innovation adoption process were also identified: sensor connectivity issues and app crashes impacted perceived usability (but forced the participants to involve in exploration and solution finding, in more intensive interaction with the technological solution than if the use flow was smooth); the complexity of multiple apps/tools overwhelmed some users (but the coaches helped participants to overcome this and go forth and learn more); and the low-literacy participants required more face-to-face support (thus increasing the social aspect of AGAPE intervention).

**Key findings** on user–technology interaction in AGAPE can be enumerated as follows:

- **Improved digital adoption and self-efficacy** (positive evolution in digital adoption profiles; personalized coaching and digital literacy support facilitated gradual uptake of digital tools and encouraged self-efficacy; repetitive exposure decreased the level of technostress).
- **Technostress declined overall, with variability across** pilot sites (the largest reduction observed among urban Romanian participants with initially higher stress levels; coaching and reassurance were perceived as supportive components although their independent effects could not be determined).
- **System Usability Scale scores (usability)** declined over time: SUS scores declined significantly over the pilot across all countries. Possible causes can be considered interface complexity, technology fatigue, rising expectations as well as the context: initial contact with coach support in person was followed by individual use with adjusted human support. The final scores showed an improvement, most probably due to the improvement of the digital skills of the participants during the study, making the same technological solution look more user-friendly due to habituation.

### 4.2. Study Limitations

Several limitations should be considered when interpreting the findings of this study. Firstly, the relatively modest sample size and the heterogeneity of the study population—including differences in age, socio-demographic characteristics, social participation and baseline levels of digital and health literacy—may limit the generalizability of the results. In addition, the intervention was highly individualized, with participants receiving different combinations of digital technologies, personalized coaching, and varying levels of technology use according to their needs and preferences. Although this person-centred approach reflects real-world practice and enhances ecological validity, it also introduces variability that may limit direct comparisons between participants and the scalability of the intervention.

The three pilot sites also differed with respect to intervention duration, recruitment procedures, participant characteristics, and technology packages. A stratified analysis by duration of exposure to AGAPE services, i.e., accounting for variability in the actual time participants spent engaging with the intervention, could have allowed for a more granular investigation of dose–response dynamics, potentially revealing more accurate between-group differences and providing deeper insight into the temporal trajectory of service efficacy across the pilot period. Because intervention duration was closely linked to pilot site and implementation context, its independent effect could not be separated from differences in baseline characteristics, living environment, coaching, and the technologies used. Accordingly, the pooled longitudinal findings should be interpreted as reflecting the overall evolution of a heterogeneous real-world intervention rather than the effect of a standardized intervention.

Furthermore, the number of participants available for analysis decreased across assessment time points because longitudinal analyses required complete paired observations and predefined follow-up intervals. Consequently, the analytical sample differed across outcomes according to the availability of valid longitudinal data. As participants with incomplete follow-up were not formally compared with those included in the final analyses, the possibility of attrition bias cannot be excluded.

Finally, the independent contribution of personalized coaching could not be quantified because coaching was delivered as facilitator in a multicomponent intervention that also included repeated technology exposure and support from family members and healthcare or social care professionals. Although qualitative feedback consistently identified the coach as an important source of motivation, technical assistance, and reassurance, these observations represent participants’ perceptions and should not be interpreted as evidence of an isolated causal effect.

Despite these limitations, the study provides valuable insights into technology acceptance, digital engagement, and behavioural adaptation among older adults and informal caregivers in real-world settings. The findings are consistent with previous research supporting the role of personalized, digitally enabled interventions in promoting active and healthy ageing.

The successful implementation of digital health solutions also depends on addressing the broader socioeconomic determinants. For many older adults, financial insecurity and unmet basic needs may take precedence over engagement with digital innovations. Consequently, socioeconomic vulnerability remains an important barrier to digital inclusion, as reflected by recent European data showing that a substantial proportion of the population continues to experience difficulties in meeting essential household needs, including adequate home heating [50].

### 4.3. Future Research

Future research should investigate more adaptive intervention models that provide access to a broader range of digital technologies while integrating structured social participation initiatives. The present findings highlight the need for comprehensive evaluation frameworks capable of capturing the broader impact of empowerment-based interventions beyond technology adoption alone.

Further studies should examine the determinants of technology adoption among older adults, including individual, contextual, motivational, technological, social, and economic factors [53].

Future research should also refine the assessment of digital skills coaching by developing outcome measures that capture both direct and indirect effects of coaching, including the contribution of human or virtual coaches, perceived social support [54], improved access to reliable health information, self-management abilities, and their wider individual, community, and societal impact [55].

Studies evaluating assistive and digital health technologies should carefully stratify target populations to better identify intervention effects across specific domains of functioning [56]. In addition to self-reported measures of digital self-efficacy, future longitudinal studies should integrate objective indicators of technology use (already available in the industry), coach observations, and individualized digital profiles, in order to better understand behavioural change. These measures should also be examined in relation to cognitive, motivational, and emotional functioning, as sustained engagement with meaningful digital activities may contribute to maintaining cognitive function in older adults as well [57]. Beyond digital skills acquisition alone, meaningful engagement with technology may enhance social connectedness [58], strengthen intergenerational relationships and community belonging [59,60,61], and reinforce an individual’s sense of coherence [62].

Finally, future studies should apply implementation science approaches to evaluate the feasibility, scalability, and long-term integration of AAL services within routine health and social care systems [63].

## 5. Conclusions

The AGAPE ecosystem represents an integrated, person-centred approach supporting active and healthy ageing by combining digital technologies with health and social care services. Implemented across diverse real-world European settings, the project was designed to address key challenges including digital exclusion, social isolation, caregiver burden, and fragmented care, while promoting independent living and quality of life.

As the fourth co-creation phase of the AGAPE project, the impact pilot study evaluated the implementation of a multicomponent intervention combining repeated use of digital technologies, personalized coaching, and support from informal and formal caregivers. Participation in the AGAPE intervention study was associated with positive longitudinal changes in technology acceptance, technology-related anxiety and technostress, digital self-efficacy, and engagement in technology-supported daily activities. The findings also highlighted the importance of contextual factors, including educational level, living environment, and social support, in shaping digital adoption among older adults.

Although the direction and magnitude of changes varied across pilot sites and individual participants, the overall findings support the feasibility and potential of implementing digitally supported, person-centred interventions across diverse real-world settings. The results suggest that an integrated ecosystem combining digital technologies with individualized and interpersonal support may facilitate technology adoption and contribute to more independent, active, and socially connected ageing. However, given the uncontrolled and multicomponent nature of the intervention, the observed changes cannot be attributed to personalized coaching or any other individual component. Future controlled studies are needed to determine the contribution of specific intervention components and to further optimize their implementation within routine health and social care services.

## Figures and Tables

**Table 1:** AGAPE technologies tested across the three pilot sites, stratified by user category.

Pilot Site/Users	Primary End Users—Older Adults	Healthcare and Social Care Professionals	Family Caregivers	Tertiary End Users—Organization Representatives
**Marradi, Italy**	Smart Band Smart Socks AGAPE Assistant IoChat	AGAPE Monitor IoChat (for the coach)	IoChat	AGAPE Eye
**Coimbra, Portugal**	Smart band or smart strap; AGAPE Assistant; IoChat; SeniorPhone	AGAPE Monitor; IoChat	IoChat	AGAPE Eye
**Bucharest, Romania**	Smart band or Smart Socks or smart straps AGAPE Assistant IoChat, Sensoria Walk	AGAPE Assistant, AGAPE Monitor, Smart Band or Smart Socks or smart straps, IoChat	AGAPE Assistant, smart band or Smart Socks or smart straps	AGAPE Monitor, AGAPE Eye

*Legend*: AGAPE Assistant = mobile app for service access; AGAPE Monitor = platform for professionals and coaches; IoChat = communication tool; AGAPE Eye = platform for tertiary users; smart band/Socks/strap = wearable devices for activity and health monitoring; Sensoria Walk = app for gait analysis; SeniorPhone = mobile interface provided to facilitate use.

**Table 2:** Participants and devices delivered during AGAPE pilot.

Site	Nr of Contacted Persons	Nr of Confirmed Users	Nr of Drop-Out	Type and Nr of Devices Provided
**Italy (FDG)**	70 OAs with their relatives; 20 FCs. 3 tertiary users.	OA: 50 IC: 10 FC: 8 TU: //	OA: 18 IC: 0 FC: 1 TU: //	Smart Socks: 6; smart band: 50; AGAPE Assistant: 50; AGAPE Monitor: 2; AGAPE Eye: //; IoChat: 18; SeniorPhone: 0.
**Portugal (CDC)**	60 OAs, 10 IC, 20 FCs and 5 tertiary users.	OA: 52 IC: 8 FC: 20 TU: 5	OA: 17 IC: 2 FC: 0 TU: 0	Smart band: 10 Smart watch: 17 AGAPE Assistant: 52 AGAPE Monitor: 20 IoChat: 28 Seniorsphone: 2
**Romania (UMFCD)**	80 OAs and their relatives; 40 FCs, 10 TUs.	OA: 49 IC: 7 FC: 19 TU: 8	0	Smart Socks: 10 pairs Smart straps: 10 pairs Smart bands: 24 AGAPE Assistant: 76 IoChat: 13 SeniorPhone: 5 AGAPE Monitor: 4 AGAPE Eye:1 Sensoria Walk app: 14

*Table 2*: Study population data from the AGAPE pilot, including the number of contacted participants, confirmed users, drop-outs, and devices provided across the three pilot sites. Legend: OA = older adult/primary end user; IC = informal caregiver/family caregiver; FC = formal caregiver/healthcare or social care professional; TU = tertiary user/organization representative; FDG = Fondazione Don Gnocchi; CDC = Cáritas Diocesana de Coimbra; UMFCD = Carol Davila University of Medicine and Pharmacy. The number of confirmed OAs reported in Table 2 refers to participants initially enrolled or confirmed at each pilot site before data cleaning and exclusion of incomplete or unusable records. Therefore, this number differs from the cleaned OA demographic dataset used for analysis (*n* = 146).

**Figure A1:**
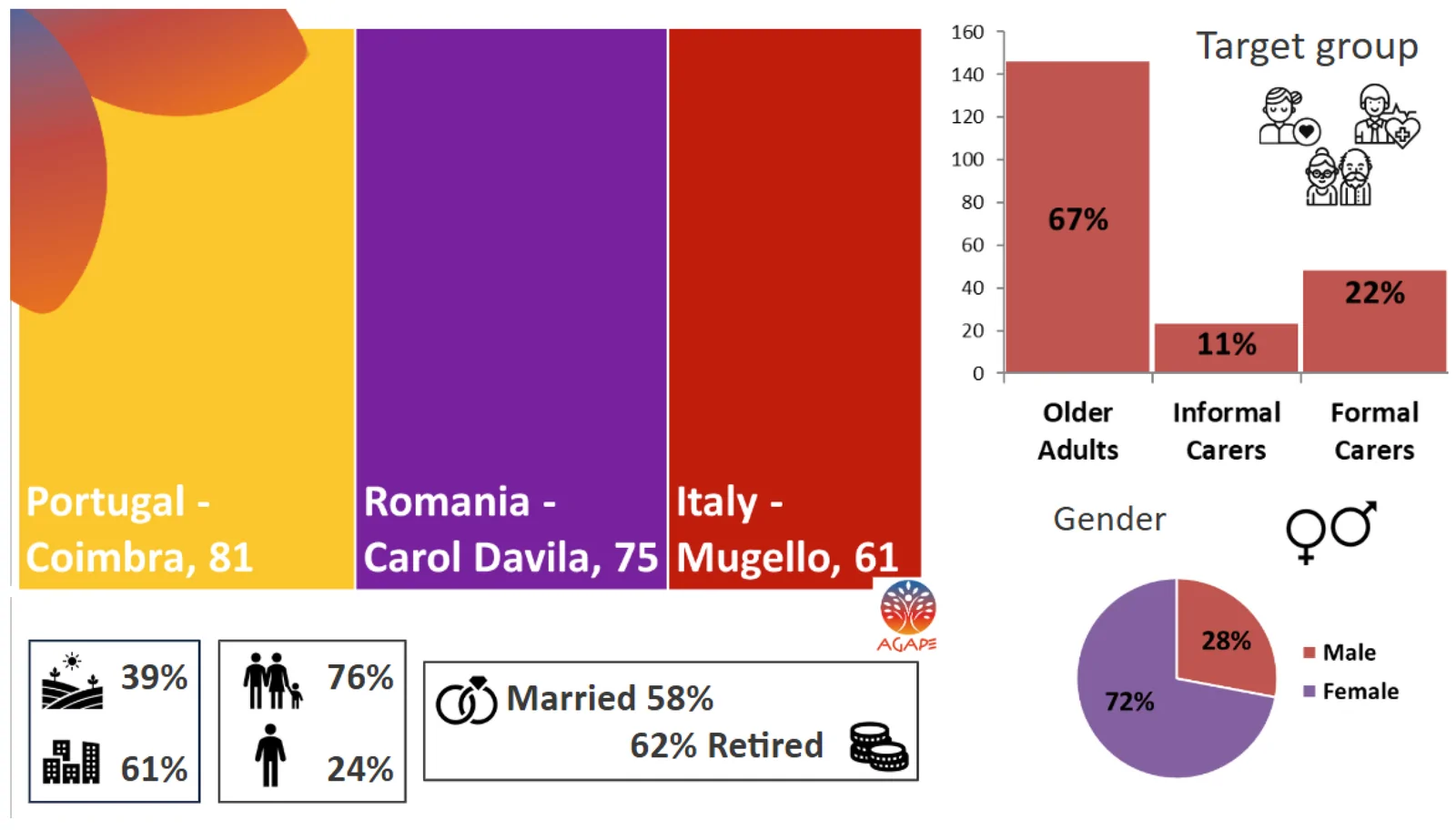
Demographic data of each pilot site (with graphs). The image includes OA’s mean age (by pilot site) and percentage of age, as well as gender.

**Figure A2:**
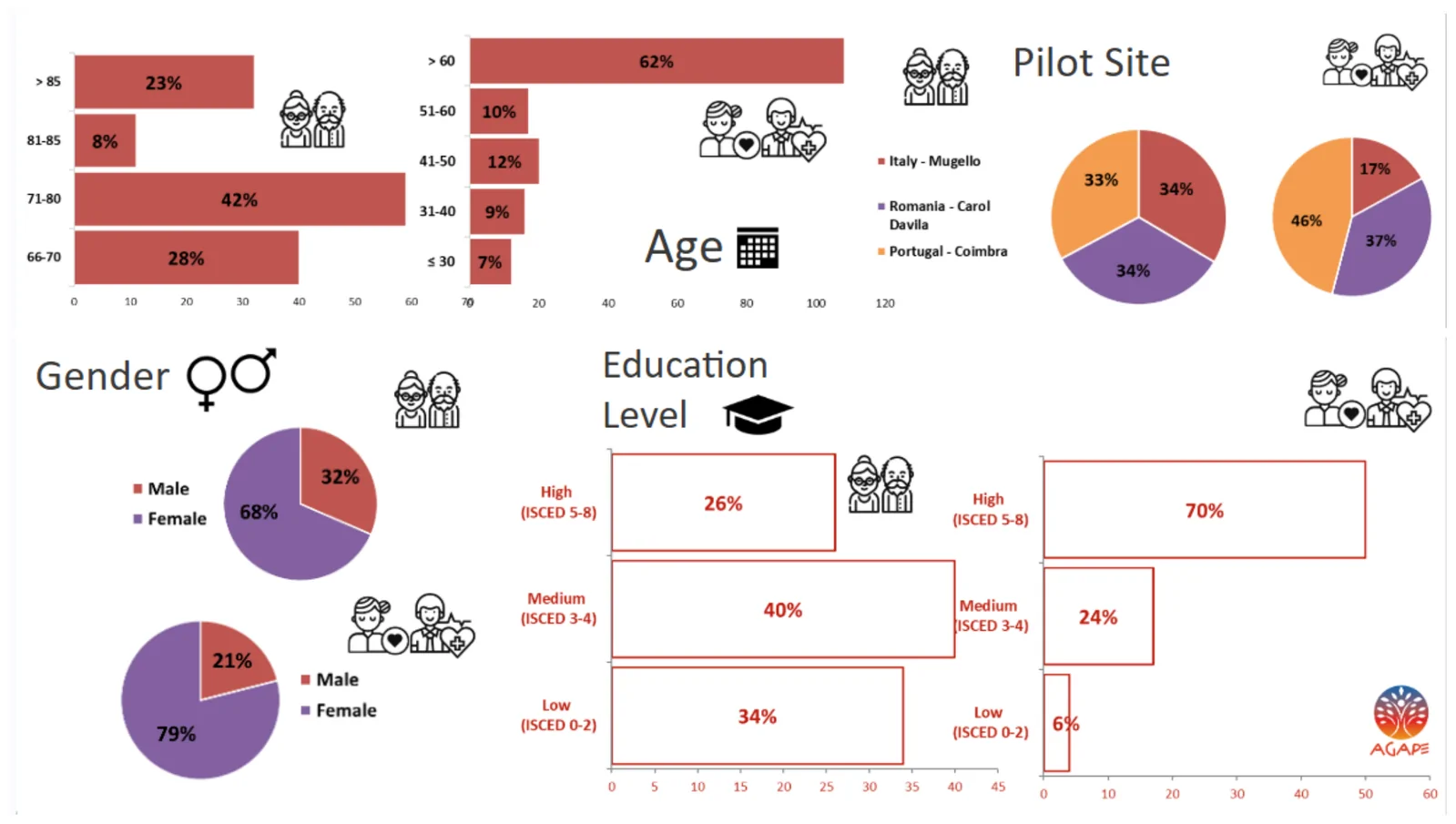
Demographic data of each pilot site (with graphs). The image includes age, gender, marital status, and education level. Percentages displayed in the figure were automatically rounded by the software and may therefore not sum exactly to 100%.

## Data Availability

The data presented in this study are available on reasonable request from the corresponding author. The data are not publicly available due to privacy.

## Appendix A. Coaches

Table A1 summarizes the roles and responsibilities of the AGAPE coaches.

**Table A1:** Roles and responsibilities of the AGAPE coach.

Training	The Coach Is Required to Be Trained on Their Responsibilities and Roles Towards Users, Taking into Account the Different Types of Users, Their Needs, and Their Goals.	
**Relationship Building** **and Support**	**User Interaction**	Coaches collaborate with users to personalize AGAPE services, ensuring that the selected tools and interventions align with each individual’s needs and preferences.
	**Regular Assistance**	Regular follow-up, performed at least once per week, is provided to encourage continued adherence to the goals established during the initial assessment.
	**Profile Adjustment**	Coaches may update user profiles and personalize intervention parameters according to adherence, user preferences, and observations collected throughout the intervention.
**Personalization** **and Adaptation**	**Adaptation to User Needs**	Coaching strategies should be adapted to each participant’s individual profile, taking into account factors such as age, health status, living arrangements, and social or cultural context.
	**Trust Building**	Developing a relationship based on trust is fundamental to effective coaching and sustained user engagement.
**Methods of** **Interaction**	**Variety of Communication Channels**	Communication between coaches and users may be conducted through multiple channels, including face-to-face meetings, telephone calls, text messaging, and video conferencing.
	**Flexible Interaction Frequency**	The coaching is individualized, with more frequent contacts provided to users requiring greater support and progressively reduced as autonomy increases.
**Encouragement** **and Motivation**	**Trust and Motivation**	Coaches are responsible for fostering motivation and trust while helping users understand the value and practical use of the technologies. Whenever appropriate, this collaborative relationship should also involve family members and healthcare or social care professionals.
	**Rewarding/Cheering Mechanism**	User engagement is reinforced through personalized motivational strategies, including positive feedback, recognition of achievements, gamification, rewards, and other incentive-based approaches.
**Tools and** **Applications**	**AGAPE Monitor Application**	The AGAPE Monitor platform serves as the coach’s main management tool for monitoring adherence, personalizing user pathways, and adapting intervention plans according to individual progress and needs.
	**Other AGAPE services**	A thorough understanding of all AGAPE services is required, together with the ability to train users, answer questions, and provide technical support whenever necessary.
**Skills and** **Qualifications**	**Empathy and Relationship Skills**	Coaches must be prepared to work effectively with older adults presenting different levels of digital and health literacy, as well as complex health conditions and care needs.
	**Familiarity with Technology**	Coaches should receive dedicated training from AGAPE experts to acquire comprehensive knowledge of the platform, its services, and the associated digital technologies.

The *coach training needs* were discovered through co-creation during the whole project and led to the development of the coach training methodology and of dedicated training materials. The coaches were also trained to be able to generate materials to impact health literacy and digital literacy of older adults and other user categories with lower knowledge and skills in these domains. The *coaching materials* and the new coaching methodology are applied in the local organizations in the pilot sites for training staff and students, in order to increase the value of the healthcare and social care staff capabilities and organization services, respectively, also in regards of geronto-psychology and behaviour change technique implementation for health and social condition improvement for their patients/clients. Coach training implementation in social care services as stand-alone service is also explored. The co-creation activities in the pre-validation and validation phases of AGAPE generated valuable knowledge regarding the real needs and attitudes of older adults, family caregivers and rehabilitation professionals in regards of digital technologies functional and non-functional requirements and objective quantitative and qualitative assessment possibilities of short and long-term digital assistive technologies’ user experience and impact. This knowledge adds to the previous knowledge of the pilot sites in smart assistive technologies and will be of great value in the designing of future assistive technologies and of future research methodologies. The common work of the research teams in all pilot sites and of the technical partner teams generated a new interdisciplinary perspective upon design thinking for well-being smart assistive technologies and strong multidomain multinational team capabilities, which can be the base for future trust, appreciation and fruitful collaboration between these teams, for future R&D projects, as well as for other initiatives, from educational to consultancy services.

## Appendix B. Population Description and Local Specificities

**Bucharest, Romania (UMFCD).** Older adults enrolled as primary users were recruited through the research team’s professional and community networks. Eligible participants were functionally independent, actively engaged in daily life, and in good physical and psychological health. Informal caregivers were recruited from participants’ family members, whereas formal caregivers were selected from the staff of the Department of Rehabilitation Medicine at Elias University Hospital, where the study was conducted. AGAPE coaches were recruited on a voluntary basis from resident physicians within the same department, and received dedicated training before participating in the intervention.

**Marradi and Palazzuolo sul Senio, Italy (FDG).** Participant recruitment was carried out in collaboration with the local Misericordia organizations, following the introduction facilitated by Società della Salute and Fondazione Don Gnocchi. As organizations responsible for many community-based elderly care and first-aid services, the Misericordia teams identified individuals who met the inclusion criteria, introduced the study, and invited interested older adults to meet with an AGAPE coach. Informal caregivers were subsequently enrolled through the participating older adults.

**Coimbra, Portugal (CDC).** Recruitment was conducted through established community organizations with long-standing links to the local older population. Participants were enrolled from one community centre, two day care centres, and the Parish Council of Santo António dos Olivais, the largest parish in Coimbra. In addition to collaborating closely with the Centre for Digital Care (CDC), the Parish Council facilitated the identification and engagement of older adults who benefited from community support services despite not being regular CDC users.

Figure A1 and Figure A2 show the demographic data for each pilot site: Portugal, Romania, and Italy.

Local specificities:

**Italy:** Due to the shortcomings created by distance and difficult access, Società della Salute del Mugello and Fondazione Don Gnocchi was prompted to dedicate the AGAPE project to these small towns. The pilot highlighted a significant issue: the fear that older adults feel when approaching technology, as well as the pressure they experience to learn how to use it, given how central digital tools have become in everyday life. Only a small number of older adults (OAs) involved in the pilot already had a relatively advanced level of digital adoption. At the beginning of the project, most participants were reluctant to engage with even the simplest features of the devices—for example, the step-counting function on smartwatches was often met with scepticism or hesitation. This initial resistance revealed a lack of confidence and a widespread perception that technology is overwhelming or “not made for them.” However, as the pilot progressed, caregivers reported a visible improvement in their family members’ quality of life. One meaningful example was the motivational impact of seeing small daily achievements—such as walking just ten more steps than the day before. These seemingly minor milestones played a big role in encouraging OAs to become more physically active, gradually integrating healthier habits into their routines. Over time, thanks to the coaching techniques, and to the continuous support provided by the AGAPE coach, many older adults began to change their mindset. They started to approach digital tools with less fear and more curiosity. Importantly, they learned to give themselves permission not to understand everything immediately, embracing a more patient, step-by-step learning process. This shift in attitude proved crucial in helping them gain confidence and autonomy in using technology, making digital tools a more natural part of their daily lives. The results of usability and technostress show a slight decrease in the scores trend, which could be attributed to the various bugs and malfunctions found in the connection between the app and devices. These technical issues may have negatively affected the user experience, increasing frustration.

**Portugal:** Coimbra was selected as the Portuguese pilot site because it hosts the Centre for Digital Care (CDC), whose long-standing experience in delivering services for older adults and close links with the local community facilitated the implementation of the study. Operating from Coimbra, CDC provides several community-based services, including community and day care centres, making the city a suitable setting for the AGAPE pilot. The majority of OAs that participated in the AGAPE pilot showed low levels of digital literacy, with only a small number of OAs showing a relatively advanced level of digital adoption. At the beginning of the pilots, most of the participants were interested in being involved in the project and in having contact with the technologies, but they always manifested difficulty in interacting with the technologies, for example, knowing how to use the AGAPE application, connecting the smart wrist band to the smartphone, among others, which led to a lack of confidence and decreased motivation at certain moments. Over time, OAs started to approach the technologies of the pilot with more curiosity and interest. With the support of the AGAPE coaches and the sessions, OAs became more autonomous over time, and it was possible to ensure that the services were adapted to individual needs, capabilities, and preferences. This personalized approach empowered OAs to take an active role in their own care and enhanced their confidence in using technology.

**Romania:** Previous evidence indicates that loneliness is highly prevalent among adults aged 65 years and older living in urban areas, including Bucharest. Approximately one-quarter of older adults reported experiencing loneliness very often, while nearly 60% stated that their daily activities were largely limited to household tasks such as cooking and cleaning. Furthermore, around 30% reported having no one to socialize with or depend on when they needed support.

## References

[1] European Parliament, Council of the European Union (2013). Regulation (EU) No 1291/2013 of the European Parliament and of the Council of 11 December 2013 Establishing Horizon 2020—The Framework Programme for Research and Innovation (2014–2020) and Repealing Decision No 1982/2006/EC. Off. J. Eur. Union.

[2] European Commission (2011). Strategic Implementation Plan for the European Innovation Partnership on Active and Healthy Ageing.

[3] Sorinmade O., Elugbadebo O., Peisah C. (2025). Ensuring Dignity in an Ageing World: Improving Care through a Human Rights Approach. Acad. Ment. Health Well-Being.

[4] Hu J., Ye M., Wan H., Leong W. (2026). The impact of digital reverse mentoring on older adults’ digital literacy: Mediating roles of self-perceptions of aging and self-efficacy. Innov. Aging.

[5] Joymangul J.S., Ciobanu I., Agnoloni F., Lampe J., Pedrini C., Pinto A., Franceschini B., Nicolas D., Tamburini E., Cecchi F. (2024). Empowering Active and Healthy Ageing: Integrating IoT and Wearable Technologies for Personalised Interventions. Appl. Sci..

[6] Ciobanu I., Irsay L., Joymangul J., Sarvari P.A., Iliescu A., Draghici R., Zamfir M., Agnoloni F., Zamfir M.V., Teodorescu M. (2024). Digital divide, smart assistive technologies and ageing people. Smart Cities Int. Conf. (SCIC) Proc..

[7] Ciobanu I., Zamfir M., Marin A.G., Zamfir M.V., Draghici R., Iliescu A., Irsay L., Berteanu M. (2023). Ageing in COVID era—Social isolation risk factors, outcomes and smart solutions. Smart Cities Int. Conf. (SCIC) Proc..

[8] Khan S., Webster S., Puxty J., Robertson M. (2026). Key Challenges and Barriers to Digital Literacy for Older Adults: Scoping Review. JMIR Aging.

[9] Yang S., Xu W., Chen K. (2025). Digital literacy and its effects on older adults’ health: Exploring mechanisms and outcomes. Front. Public Health.

[10] Tirado-Morueta R., Rodríguez-Alarcón L., Contreras Pulido P., Illanes-Segura R. (2025). Contextualizing Digital Literacy for Older Adults: A Study Based on Focus Groups and Guiding Theory. Wellbeing Space Soc..

[11] Pedrini C., Szczepanska M.A., Ciobanu I., Tazzari S., Pinto A., Joymangul J., Tamburini E., Dionisio P., Pinna S., Longo D. (2025). Developing an ecosystem of advanced digital services for healthy ageing by a co-creation methodology: Preliminary results of the AGAPE project. J. Public Health Res..

[12] Brooke J., Jordan P.W., Thomas B., Weerdmeester B.A., McClelland I.L. (1996). SUS: A “Quick and Dirty” Usability Scale. Usability Evaluation in Industry.

[13] Davis F.D. (1989). Perceived Usefulness, Perceived Ease of Use, and User Acceptance of Information Technology. MIS Q..

[14] Tarafdar M., Tu Q., Ragu-Nathan T.S., Ragu-Nathan B.S. (2007). The Impact of Technostress on Role Stress and Productivity. J. Manag. Inf. Syst..

[15] Herdman M., Gudex C., Lloyd A., Janssen M.F., Kind P., Parkin D., Bonsel G., Badia X. (2011). Development and Preliminary Testing of the New Five-Level Version of EQ-5D (EQ-5D-5L). Qual. Life Res..

[16] Brouwer W.B.F., van Exel N.J.A., van Gorp B., Redekop W.K. (2006). The CarerQol Instrument: A New Instrument to Measure Care-Related Quality of Life of Informal Caregivers for Use in Economic Evaluations. Qual. Life Res..

[17] Russell D.W. (1996). UCLA Loneliness Scale (Version 3): Reliability, Validity, and Factor Structure. J. Personal. Assess..

[18] Venkatesh V., Morris M.G., Davis G.B., Davis F.D. (2003). User Acceptance of Information Technology: Toward a Unified View. MIS Q..

[19] Nugent C., Cleland I., Nugent L., Estevez M.E., Lendinez A.M., Craig D., Agnoloni F., Tamburini E., Bravo J., Urzáiz G. (2023). Using Generative AI to Assist with Technology Adoption Assessment. Proceedings of the 15th International Conference on Ubiquitous Computing & Ambient Intelligence (UCAmI 2023).

[20] Liu Y., Lu X., Zhao G., Li C., Shi J. (2022). Adoption of Mobile Health Services Using the Unified Theory of Acceptance and Use of Technology Model: Self-Efficacy and Privacy Concerns. Front. Psychol..

[21] Rouidi M., Abd Elmajid E., Hamdoune A., Choujtani K., Chati A. (2022). TAM UTAUT and the Acceptance of Remote Healthcare Technologies by Healthcare Professionals: A Systematic Review. Inform. Med. Unlocked.

[22] Bates D., Kliegl R., Vasishth S., Baayen H. (2015). Parsimonious Mixed Models. arXiv.

[23] Laird N.M., Ware J.H. (1982). Random-effects models for longitudinal data. Biometrics.

[24] Thorndike R.L. (1953). Who Belongs in the Family?. Psychometrika.

[25] WHO Ageing and Health. https://www.who.int/news-room/fact-sheets/detail/ageing-and-health.

[26] Florez-Revuelta F., Ake-Kob A., Climent-Perez P., Coelho P., Colonna L., Dahabiyeh L., Dantas C., Dogru-Huzmeli E., Ekenel H.K., Jevremovic A. (2024). 50 Questions on Active Assisted Living Technologies. Global Edition.

[27] Bouma H. (1998). Gerontechnology: Emerging Technologies and Their Impact on Aging in Society. Stud. Health Technol. Inform..

[28] Chen Q., Li Z., Zhao M., Huang X. (2025). Technology anxiety in elderly patients with chronic co-morbidities: A latent profile analysis. BMC Public Health.

[29] Søraa R.A., Nyvoll P., Tøndel G., Fosch-Villaronga E., Serrano J.A. (2021). The social dimension of domesticating technology: Interactions between older adults, caregivers, and robots in the home. Technol. Forecast. Soc. Change.

[30] Chen S. (2024). Age-appropriate design of smart senior care product APP interface based on deep learning. Heliyon.

[31] Wildenbos G.A., Jaspers M.W.M., Schijven M.P., Dusseljee-Peute L.W. (2019). Mobile health for older adult patients: Using an aging barriers framework to classify usability problems. Int. J. Med. Inform..

[32] Selwyn N., Gorard S., Furlong J. (2006). Adult Learning in the Digital Age: Information Technology and the Learning Society.

[33] Liu T., Luo Y.T., Pang P.C. (2025). Digital technologies-enhanced older adults health management: Developing a five-dimensional extension of social learning theory for community settings. Front. Public Health.

[34] Janböcke S., Ogawa T., Langendorf J., Kobayashi K., Browne R., Wieching R., Taki Y. (2023). Human Coach Technology Reactance Factors and their Influence on End-Users’ Acceptance of e-Health Applications. Int. J. Adv. Comput. Sci. Appl..

[35] Bevilacqua R., Casaccia S., Cortellessa G., Astell A., Lattanzio F., Corsonello A., D’Ascoli P., Paolini S., Di Rosa M., Rossi L. (2020). Coaching Through Technology: A Systematic Review into Efficacy and Effectiveness for the Ageing Population. Int. J. Environ. Res. Public Health.

[36] Santini S., Fabbietti P., Galassi F., Merizzi A., Kropf J., Hungerländer N., Stara V. (2023). The Impact of Digital Coaching Intervention for Improving Healthy Ageing Dimensions among Older Adults during Their Transition from Work to Retirement. Int. J. Environ. Res. Public Health.

[37] Yang H.J., Lee J.H., Lee W. (2025). Factors Influencing Health Care Technology Acceptance in Older Adults Based on the Technology Acceptance Model and the Unified Theory of Acceptance and Use of Technology: Meta-Analysis. J. Med. Internet Res..

[38] Nimrod G. (2025). Technostress in later life: A multinational study. Educ. Gerontol..

[39] Van Baelen F., De Regge M., Larivière B., Verleye K., Eeckloo K. (2025). The impact of family members on aging persons’ technology use intentions. Heliyon.

[40] Takano E., Maruyama H., Takahashi T., Mori K., Nishiyori K., Morita Y., Fukuda T., Kondo I., Ishibashi Y. (2023). User Experience of Older People While Using Digital Health Technologies: A Systematic Review. Appl. Sci..

[41] Eurostat Individuals’ Level of Digital Skills (From 2021 Onwards). https://ec.europa.eu/eurostat/databrowser/view/isoc_sk_dskl_i21__custom_9882764/bookmark/table?lang=en&bookmarkId=bd5414aa-dddd-4a29-bdd1-22dbee6a1342&c=1707988291329.

[42] Eurostat Digital Skills in 2023: Impact of Education and Age. https://ec.europa.eu/eurostat/web/products-eurostat-news/w/ddn-20240222-1.

[43] Hertzum M. (2026). System Usability Scale: A Meta-Analysis of How SUS Relates to Workload, Task Time, and Error Rate. Int. J. Hum.-Comput. Interact..

[44] Borawska A., Mateja A., Rodrigues da Silva A., Mira da Silva M., Estima J., Barry C., Lang M., Linger H., Schneider C. (2024). Unveiling the User Experience: A Synthesis of Cognitive Neuroscience Methods in Digital Product Design. Advances in Information Systems Development.

[45] Rašticová M., Šácha J., Lakomý M., Mishra P.K. (2025). Technostress Among Older Workers: A Central European Perspective. Psychol. Res. Behav. Manag..

[46] Pani J., Lorusso L., Toccafondi L., D’Onofrio G., Ciccone F., Russo S., Giuliani F., Sancarlo D., Calamida N., Vignani G. (2024). How Time, Living Situation, and Stress Related to Technology Influence User Acceptance and Usability of a Socialization Service for Older Adults and Their Formal and Informal Caregivers: Six-Month Pilot Study. JMIR Aging.

[47] Graham S.A., Stein N., Shemaj F., Branch O.H., Paruthi J., Kanick S.C. (2021). Older Adults Engage with Personalized Digital Coaching Programs at Rates That Exceed Those of Younger Adults. Front. Digit. Health.

[48] Niu H.J., Li M.H., Hsieh F.Y., Yu C.C., Lin C.T. (2026). From Digital Anxiety to Empowerment in Older Adults: Cross-Sectional Survey Study on Psychosocial Drivers of Digital Literacy. JMIR Aging.

[49] An J., Zhu X., Wan K., Xiang Z., Shi Z., An J., Huang W. (2024). Older adults’ self-perception, technology anxiety, and intention to use digital public services. BMC Public Health.

[50] EC Eurostat Housing in Europe, 2025 Edition. https://ec.europa.eu/eurostat/web/interactive-publications/housing-2025.

[51] SeniorsNet Federation News. Niciodată Singur and Their Study on Loneliness Among Older People. https://seniorinet.ro/niciodata-singur-and-their-study-on-loneliness-among-older-people/?lang=en.

[52] Pfeffer R.I., Kurosaki T.T., Harrah C.H., Chance J.M., Filos S. (1982). Measurement of functional activities in older adults in the community. J. Gerontol..

[53] Pani J., Fiorini L., Rovini E., Giuliani F., Lorusso L., Russo S., do Nascimento Teixeira Fernandes A.C., de Oliveira A.G., Pitarma E., Rodrigues Â. (2026). Identifying and evaluating factors influencing technology adoption: A multi-pilot study within the Pharaon project. Int. J. Med. Inform..

[54] Czaja S.J., Boot W.R., Charness N., Rogers W.A., Sharit J. (2018). Improving social support for older adults through technology: Findings from the PRISM randomized controlled trial. Gerontologist.

[55] Hwang M., Park Y.-H., Lee S., Park G.E. (2025). Effectiveness of a digital health coaching self-management program for older adults living alone with multiple chronic conditions: A randomized controlled trial. Geriatr. Nurs..

[56] Mannheim I., Schwartz E., Xi W., Buttigieg S.C., McDonnell-Naughton M., Wouters E.J.M., van Zaalen Y. (2019). Inclusion of Older Adults in the Research and Design of Digital Technology. Int. J. Environ. Res. Public Health.

[57] Chan M.Y., Haber S., Drew L.M., Park D.C. (2016). Training older adults to use tablet computers: Does it enhance cognitive function?. Gerontologist.

[58] Kebede A.S., Ozolins L., Holst H., Galvin K. (2022). Digital engagement of older adults: Scoping review. J. Med. Internet Res..

[59] Wu J., Siu K.W.M., Zhang L. (2023). Intergenerational Integration in Community Building to Improve the Mental Health of Residents—A Case Study of Public Space. Behav. Sci..

[60] Sun M., Zhao Z., Liu H. (2025). Fostering intergenerational communication through games: An exploratory study on metaphorical life games in family settings. Front. Public Health.

[61] Petersen B., Khalili-Mahani N., Murphy C., Sawchuk K., Phillips N., Li K.Z.H., Hebblethwaite S. (2023). The Association between Information and Communication Technologies, Loneliness and Social Connectedness: A Scoping Review. Front. Psychol..

[62] Meier Magistretti C., Mittelmark M.B., Bauer G.F., Vaandrager L., Pelikan J.M., Sagy S., Eriksson M., Lindström B., Magistretti C.M. (2022). The Sense of Coherence in the Life Course. The Handbook of Salutogenesis.

[63] Resnick B., Boltz M., Galik E., Fix S., Holmes S., Zhu S., Barr E. (2021). Testing the Implementation of Function-focused Care in Assisted Living Settings. J. Am. Med. Dir. Assoc..

